# Trajectory of Cognitive Decline Before and After Stroke in 14 Population Cohorts

**DOI:** 10.1001/jamanetworkopen.2024.37133

**Published:** 2024-10-02

**Authors:** Jessica W. Lo, John D. Crawford, Darren M. Lipnicki, Richard B. Lipton, Mindy J. Katz, Pierre-Marie Preux, Maëlenn Guerchet, Eleonora d’Orsi, Anna Quialheiro, Cassiano Ricardo Rech, Karen Ritchie, Ingmar Skoog, Jenna Najar, Therese Rydberg Sterner, Elena Rolandi, Annalisa Davin, Michele Rossi, Steffi G. Riedel-Heller, Alexander Pabst, Susanne Röhr, Mary Ganguli, Erin Jacobsen, Beth E. Snitz, Kaarin J. Anstey, Allison E. Aiello, Henry Brodaty, Nicole A. Kochan, Yen-Ching Chen, Jen-Hau Chen, Pascual Sanchez-Juan, Teodoro del Ser, Meritxell Valentí, Antonio Lobo, Concepción De-la-Cámara, Elena Lobo, Perminder S. Sachdev

**Affiliations:** 1 Centre for Healthy Brain Ageing, Discipline of Psychiatry and Mental Health, School of Clinical Medicine, University of New South Wales, Sydney, Australia; 2 Saul B. Korey Department of Neurology, Albert Einstein College of Medicine, Bronx, New York; 3 Inserm U1094, IRD UMR270, Univ. Limoges, CHU Limoges, EpiMaCT - Epidemiology of chronic diseases in tropical zone, Institute of Epidemiology and Tropical Neurology, OmegaHealth, Limoges, France; 4 Laboratory of Chronic and Neurological Diseases Epidemiology, Faculty of Health Sciences, University of Abomey-Calavi, Cotonou, Benin; 5 Federal University of Santa Catarina, Trindade University Campus, Florianópolis, Santa Catarina, Brazil; 6 IA&Saúde—The Artificial Intelligence and Health Research Unit, Polytechnic University of Health, CESPU, Portugal; 7 Department of Physical Education, Federal University of Santa Catarina, Florianópolis, Santa Catarina, Brazil; 8 Inserm U1061: Neuropsychiatrie Hôpital La Colombière, BP34493, Montpellier, France; 9 Section of Psychiatry and Neurochemistry, Institute of Neuroscience and Physiology, Sahlgrenska Academy, University of Gothenburg, Gothenburg, Sweden; 10 Region Västra Götaland, Sahlgrenska University Hospital, Psychiatry, Cognition and Old Age Psychiatry Clinic, Gothenburg, Sweden; 11 Department of Psychotic Disorders, Region Västra Götaland, Sahlgrenska University Hospital, Gothenburg, Sweden; 12 Section Genomics of Neurdegenerative Diseases and Aging, Department of Human Genetics Amsterdam UMC, Vrije Universiteit Amsterdam, Amsterdam UMC, Amsterdam, the Netherlands; 13 Aging Research Center, Department of Neurobiology, Care Sciences and Society, Karolinska Institutet and Stockholm University, Stockholm, Sweden; 14 Neuropsychiatric Epidemiology Unit, Department of Psychiatry and Neurochemistry, Institute of Neuroscience and Physiology, The Sahlgrenska Academy, Centre for Ageing and Health, University of Gothenburg, Gothenburg, Sweden; 15 Golgi Cenci Foundation, Abbiategrasso, Italy; 16 Department of Brain and Behavioural Sciences, University of Pavia, Pavia, Italy; 17 Faculty of Medicine, Institute of Social Medicine, Occupational Health and Public Health, University of Leipzig, Leipzig, Germany; 18 School of Psychology, Massey University, Albany Campus, Auckland, Aotearoa, New Zealand; 19 Global Brain Health Institute, Trinity College Dublin, Dublin, Ireland; 20 Departments of Psychiatry, Neurology, and Epidemiology, School of Medicine and School of Public Health, University of Pittsburgh, Pittsburgh, Pennsylvania; 21 Department of Psychiatry, University of Pittsburgh School of Medicine, Pittsburgh, Pennsylvania; 22 Department of Neurology, University of Pittsburgh School of Medicine, Pittsburgh, Pennsylvania; 23 Ageing Futures Institute, University of New South Wales, Sydney, Australia; 24 Neuroscience Research Australia, Sydney, Australia; 25 School of Psychology, University of New South Wales, Sydney, Australia; 26 Department of Epidemiology and Robert N. Butler Columbia Aging Center, Mailman School of Public Health, Columbia University, New York, New York; 27 Institute of Epidemiology and Preventive Medicine, College of Public Health, National Taiwan University, Taipei, Taiwan; 28 Master Program of Statistics, National Taiwan University, Taipei, Taiwan; 29 Department of Geriatrics and Gerontology, National Taiwan University Hospital, Taipei, Taiwan; 30 Department of Internal Medicine, National Taiwan University Hospital, Taipei, Taiwan; 31 Alzheimer’s Centre Reina Sofia-CIEN Foundation-ISCIII, 28031, Madrid, Spain; 32 Department of Medicine and Psychiatry, Universidad de Zaragoza, Zaragoza, Spain; 33 Instituto de Investigación Sanitaria Aragón, Zaragoza, Spain; 34 Centro de Investigación Biomédica en Red de Salud Mental, Madrid, Spain; 35 Department of Preventive Medicine and Public Health, Universidad de Zaragoza, Zaragoza, Spain

## Abstract

**Importance:**

Poststroke cognitive impairment is common, but the cognitive trajectory following a first stroke, relative to prestroke cognitive function, remains unclear.

**Objective:**

To map the trajectory of cognitive function before any stroke and after stroke in global cognition and in 4 cognitive domains, as well as to compare the cognitive trajectory prestroke in stroke survivors with the trajectory of individuals without incident stroke over follow-up.

**Design, Setting, and Participants:**

The study used harmonized and pooled data from 14 population-based cohort studies included in the Cohort Studies of Memory in an International Consortium collaboration. These studies were conducted from 1993 to 2019 across 11 countries among community-dwelling older adults without a history of stroke or dementia. For this study, linear mixed-effects models were used to estimate trajectories of cognitive function poststroke relative to a stroke-free cognitive trajectory. The full model adjusted for demographic and vascular risk factors. Data were analyzed from July 2022 to March 2024.

**Exposure:**

Incident stroke.

**Main outcomes and measures:**

The primary outcome was global cognition, defined as the standardized average of 4 cognitive domains (language, memory, processing speed, and executive function). Cognitive domain scores were formed by selecting the most commonly administered test within each domain and standardizing the scores.

**Results:**

The study included 20 860 participants (12 261 [58.8%] female) with a mean (SD) age of 72.9 (8.0) years and follow-up of 7.51 (4.2) years. Incident stroke was associated with a substantial acute decline in global cognition (−0.25 SD; 95% CI, −0.33 to −0.17 SD), the Mini-Mental State Examination, and all cognitive domains (ranging from −0.17 SD to −0.22 SD), as well as accelerated decline in global cognition (−0.038 SD per year; 95% CI, −0.057 to −0.019 SD per year) and all domains except memory (ranging from −0.020 to −0.055 SD per year), relative to a stroke-free cognitive trajectory. There was no significant difference in prestroke slope in stroke survivors compared with the rate of decline in individuals without stroke in all cognitive measures. The mean rate of decline without a previous stroke was −0.049 SD per year (95% CI, −0.051 to −0.047 SD) in global cognition.

**Conclusions and relevance:**

In this cohort study using pooled data from 14 cohorts, incident stroke was associated with acute and accelerated long-term cognitive decline in older stroke survivors.

## Key Points

**Question:**

What is the outcome of a first stroke on cognitive function?

**Findings:**

In this cohort study of 14 international cohorts of older adults, stroke was associated with a significant acute decline of 0.25 SD in global cognition and a small but significant acceleration in the rate of decline of −0.038 SD per year compared with decline without a previous stroke (−0.049 SD per year). The cognitive performance of stroke survivors before stroke was similar to that of individuals without incident stroke over follow-up.

**Meaning:**

These findings suggest incident stroke is associated with acute and accelerated long-term cognitive decline in older adults.

## Teaser

This cohort study evaluates the trajectory of cognitive function before any stroke and after stroke in global cognition and in 4 cognitive domains.

## Introduction

Stroke is a leading cause of disability and dementia worldwide, with projections suggesting a continued rise in its prevalence and burden.^1^ Recent studies have shown that cognitive impairment is highly prevalent after stroke, with cognitive deficits present in over a third of stroke survivors.^2,3^ However, the precise impact of stroke on the trajectory of cognitive function remains unclear. Previous studies, primarily hospital-based, have been unable to account for prestroke cognitive performance, and several population-based studies examining prestroke and poststroke cognitive function reported conflicting findings,^4,5,6,7,8^ likely due to variations in study design, sample characteristics, and statistical techniques.

This study aimed to address these inconsistencies by mapping the trajectory of cognitive function after stroke relative to the cognitive trajectory without a previous stroke using harmonized data from diverse population cohorts from the Cohort Studies of Memory in an International Consortium (COSMIC).^9^ Secondary aims were to compare the cognitive trajectory in the years preceding incident stroke with the cognitive performance of those who remained stroke-free over follow-up and to identify factors associated with risk contributing to changes in poststroke cognitive trajectory.

## Methods

This cohort study was approved by the University of New South Wales human research ethics committee. Each study had independent approval from its regional ethics board, and their participants provided informed consent. The Strengthening the Reporting of Observational Studies in Epidemiology (STROBE) reporting guideline was used for reporting.^10^

### Sample

COSMIC member studies are population-based longitudinal studies of older individuals. We included 14 studies conducted from 1993 to 2019 meeting the following criteria: (1) conducted at least 2 follow-up neuropsychological assessments and (2) collected data on interval stroke. Follow-up durations range from 3 to 17 years across cohorts. Participants with a history of stroke or dementia at baseline (criteria provided in eTable 1 in Supplement 1) were excluded from the analyses. Table 1 and eTable 1 in Supplement 1 summarize each study.^11,12,13,14,15,16,17,18,19,20,21,22,23,24^

**Table 1.** Study and Participant Characteristics

Study	No. of participants^a^	Location	Year study started	Max follow-up duration, y (No. of waves)	Main ethnic and racial group^b^	Publication, y
Einstein Aging Study (EAS)	1915	New York, US	1993	17 (18)	67.8% White, 25.9% Black, and 4.7% Hispanic	Katz et al,^11^ 2011
Epidemiology of Dementia in Central Africa (EPIDEMCA)	448	Republic of Congo	2011	3 (4)	African	Guerchet et al,^12^ 2014
EpiFloripa Aging Study (EpiFloripa)	1054	Florianópolis, Brazil	2009	10 (3)	Brazilian	Schneider et al,^13^ 2017
Etude SanteÂ Psychologique et Traitement (ESPRIT)	2098	Montpellier, France	1999	17 (7)	White	Ritchie et al,^14^ 2010
Gothenburg H70 Birth Cohort Studies (H70 study)	550	Gothenburg, Sweden	1971	15 (4)	White	Rydberg Sterner et al,^15^ 2019
Invecchiamento Cerebrale in Abbiategrasso (Invece.Ab)	1082	Abbiategrasso, Italy	2010	8 (4)	White	Guaita et al,^16^ 2013
Leipzig Longitudinal Study of the Aged (LEILA75+)	878	Leipzig, Germany	1997	17 (7)	White	Reidel-Heller et al,^17^ 2001
Monongahela-Youghiogheny Healthy Aging Team (MYHAT)	1808	Pennsylvania, US	2006	13 (13)	White	Ganguli et al,^18^ 2009
Personality and Total Health Through Life Project (PATH)	2420	Canberra, Australia	2001	14 (4)	White	Anstey et al,^19^ 2012
Sacramento Area Latino Study on Aging (SALSA)	1565	Sacramento Valley, California, US	1998	9 (7)	Mexican	Haan et al,^20^ 2003
Sydney Memory and Aging Study (Sydney MAS)	996	Sydney, Australia	2005	13 (7)	White	Sachdev et al,^21^ 2010
Taiwan Initiative for Geriatric Epidemiological Research (TIGER)	566	Taipei, Taiwan	2011	7 (4)	Chinese	Lin et al,^22^ 2021
Vallecas Project (Vallecas)	1103	Madrid, Spain	2011	8 (9)	White	Olazarán et al,^23^ 2015
Zaragoza Dementia Depression Project (ZARADEMP)	4377	Zaragoza, Spain	1994	14 (4)	White	Lobo et al,^24^ 2005

^a^ Sample size for the present project, which included participants with baseline assessment who were stroke-free and without a dementia diagnosis.

^b^ Ethnic and racial groups were self-identified or as determined by the study investigator as the predominant ethnic and racial group in the cohort.

### Stroke and Baseline Factors

Stroke was self-reported in all studies except 2,^15,16^ where the information came from an inpatient register or via examination (eTable 2 in Supplement 1). Year and month of stroke was recorded or approximated by the midpoint between 2 assessments (eTable 2 in Supplement 1). Demographic and medical history data were harmonized as per previous COSMIC projects (see eTable 3 and eTable 4 in Supplement 1).^25,26,27^ Baseline factors considered were age; sex; education in years; race, ethnicity, or nationality (self-identified or investigator-observed by the investigators in each study); study entry period (by decade); apolipoprotein E ε4 allele (APOE4) carrier; blood pressure; body mass index, smoking (ever); alcohol use; physical activity; depression; diabetes; hypertension; high cholesterol; and cardiovascular disease (CVD). Race, ethnicity, and nationality were included in the analyses due to reported differences in stroke outcomes across racial groups. Table 2 lists the categories for each harmonized variable; eTable 3 and eTable 5 in Supplement 1 provide the criteria and levels of missing data.

**Table 2.** Baseline Characteristics by Participants With and Without Incident Stroke

Variable	Participants, No. (%)		*P* value	Cohen *d* or Cohen *h*^a^
	Incident stroke (n = 1041)	No incident stroke (n = 19 819)		
Demographics				
Age at baseline, mean (SD), y	73.9 (7.6)	72.9 (8.0)	<.001	0.13
Study entry period				
Before 1990	40 (3.8)	194 (1.0)	<.001	0.16^b^
1990-1999	552 (53.0)	8883 (44.8)		
2000-2009	370 (35.5)	8436 (42.6)		
After 2010	79 (7.6)	2306 (11.6)		
Sex				
Female	602 (57.8)	11 659 (58.8)	.52	0.02
Male	439 (42.2)	8160 (41.2)		
Education, mean (SD), y	9.1 (4.9)	10.1 (4.8)	<.001	0.18
Race, ethnicity, or nationality				
African	34 (3.3)	414 (2.1)	<.001	0.15^b^
Asian (90% Chinese)	7 (0.7)	626 (3.2)		
Black (US)	12 (1.2)	562 (2.8)		
Brazilian	86 (8.3)	968 (4.9)		
Hispanic (US)	3 (0.3)	100 (0.5)		
Mexican	145 (13.9)	1420 (7.2)		
White	754 (72.4)	15 654 (79.0)		
Other	0	75 (0.4)		
Vascular risk factors^c^				
Body mass index, mean (SD)^d^	27.6 (5.4)	26.9 (5.1)	<.001	0.15
APOE ε4 carrier	135 (20.3)	2624 (21.1)	.63	0.02
Blood pressure, mean (SD)				
Systolic	146.1 (21.7)	140.6 (20.0)	<.001	0.27
Diastolic	81.5 (13.1)	79.7 (11.6)	<.001	0.16
Diabetes	265 (25.6)	3324 (16.9)	<.001	0.21
Hypertension	795 (76.7)	13578 (68.9)	<.001	0.18
High cholesterol	342 (40.6)	5974 (35.0)	.001	0.12
Cardiovascular disease	215 (23.6)	3223 (17.1)	<.001	0.16
Smoker (ever)	463 (44.7)	8423 (42.7)	.19	0.04
Alcohol use				
None/minimal	565 (56.2)	10361 (55.5)	.39	0.01^b^
1 drink/wk	148 (14.7)	3051 (16.3)		
≥2 drink/wk	292 (29.1)	5258 (28.2)		
Physical activity				
Minimal	185 (28.0)	3039 (24.1)	.13	0.03^b^
Moderate	344 (52.5)	6694 (54.1)		
Vigorous	128 (19.5)	2743 (21.8)		
Depression	245 (24.4)	3665 (19.6)	<.001	0.11
Baseline cognitive scores, mean (SD)^c^				
MMSE	27.5 (2.6)	27.4 (2.4)	.81	0.09
Global cognition	−0.07 (1.03)	0.002 (1.01)	.18	0.08
Processing speed	−0.10 (0.99)	0.002 (1.06)	.70	0.02
Memory	−0.18 (1.07)	−0.05 (1.09)	<.001	0.12
Language	0.06 (1.05)	0.07 (1.08)	.63	0.01
Executive function	0.03 (1.12)	0.13 (1.09)	.048	0.09

Abbreviation: MMSE, Mini-Mental State Examination.

^a^ For both Cohen *d* and Cohen *h*, values of 0.2, 0.5, and 0.8 are taken to represent small, medium, and large differences between groups in means or proportions.

^b^ Cohen h was calculated for the most common category.

^c^ All vascular risk factors and cognitive scores have missing data. Refer to eTable 5 in Supplement 1 for the number of missing values for each variable.

^d^ Calculated as weight in kilograms divided by height in meters squared.

### Cognitive Tests

Based on previous COSMIC work,^25,26^ domain scores were calculated by selecting the most common test administered in each cognitive domain (memory, processing speed, language, and executive function). Domain and Mini-Mental State Examination (MMSE) scores were standardized using the demographic category-centered method^28^ based on the average person in the combined sample (age 73 years; male; education 10 years). See eTable 6 in Supplement 1 for the tests used in each domain from each study. Global cognition was the standardized mean of the *z* scores from at least 3 cognitive domains.

### Statistical Analysis

Participants were categorized into stroke and no-stroke groups based on whether they experienced an interval stroke during follow-up. Baseline characteristics were compared between the groups using *t* test or χ^2^ tests, and the magnitude of differences assessed using Cohen *d* or Cohen *h* as appropriate.

Regression discontinuity design^29^ with 2 sequential linear mixed-effects functions was used to model the cognitive trajectory poststroke relative to the trajectory over which participants were stroke-free.^4,5,6^ The basic model included time in study (TIS), time since stroke (TSS), and stroke (time-varying variable changing from 0 to 1 at time of stroke). The model coefficient for TIS quantifies the rate of decline (slope) for all individuals over the period without stroke. The TSS coefficient estimates the difference in slope poststroke relative to TIS and can be interpreted as the long-term outcome of stroke on the rate of cognitive decline. The stroke coefficient quantifies the difference in level of cognitive function between the stroke-free and poststroke trajectories at time of stroke (TSS = 0) and can be interpreted as the acute outcome of stroke on cognition level.

Quadratic terms were included to examine nonlinear trends and retained if significant at *P* < .05. Random intercepts were included to accommodate correlation of cognitive measures within participants over time and between studies.^30^ The adjusted model additionally included age, sex, education, and baseline factors that were *P* < .10 when examined individually in the basic model. Missing covariates in the pooled sample were imputed using multiple imputation with chain equations (eMethods in Supplement 1).^31^ Global cognition was the primary outcome, and the 4 domain scores and MMSE were secondary outcomes. For 0.2% of participants with 2 incident strokes, we censored cognitive assessments after their second stroke. Trajectory plots were constructed using projected values of cognition calculated for the means of included covariates. The analysis was performed first in the whole sample, and then separately in the stroke and no-stroke groups. See eTable 7 in Supplement 1 for detailed interpretation of the model coefficients.

#### Secondary Aims and Sensitivity Analyses

Differences in cognitive trajectories between the groups were examined by including a group variable and its interaction with TIS in the adjusted model, with TIS restricted to before stroke. We examined factors associated with poststroke cognitive trajectory by including interaction terms of TIS, TSS, and stroke with demographic and vascular factors associated with risk separately in the adjusted model with global cognition as the outcome.

Three sensitivity analyses were conducted for the key analysis: (1) including only participants with complete data; (2) excluding cognitive assessments within 1 year of an incident stroke, given instability in cognition up to 1 year poststroke^32^; and (3) excluding studies with more than 50% loss to follow-up at the final wave. Analyses were performed with Stata version 18.0 (StataCorp) from July 2022 to March 2024.

## Results

### Summary Statistics

The mean (SD) age of the full sample of 20 860 participants was 72.9 (8.0) years, with 12 261 (58.8%) female, 448 (2.2%) African, 633 (3.0%) Asian, 574 (2.8%) Black, 1054 (5.1%) Brazilian, 103 (0.5%) Hispanic, 1565 (7.5%) Mexican, and 16 408 (78.7%) White participants. The mean education level was 10.1 (4.8) years. A total of 1041 (5.0%) experienced a first incident stroke, occurring a mean (SD) of 4.55 (3.7) years after study entry at a mean (SD) age of 79.5 (7.5) years. A total of 8573 participants (41.1%) were followed up until the last assessment. Follow-up durations ranged from 3 to 17 years (Table 1; eFigure 1 in Supplement 1), with a mean (SD) duration of 7.51 (4.2) years. Participant characteristics in each study are detailed in eTable 8 and eTable 9 in Supplement 1. Participants who dropped out compared with those assessed until study end were significantly older, had fewer years of education, and had higher proportions with vascular risk factors at baseline (eTable 10 in Supplement 1), though Cohen *d* and *h* values suggest that apart from age, these differences were small.

Baseline characteristics between the stroke and no-stroke groups differed significantly, including age, education, and proportions of participants with vascular risk factors. However, the magnitude of differences between the 2 groups was small, with Cohen *d* and *h* values less than 0.28 (Table 2).

### Trajectory of Global Cognition Without Previous Stroke and After Stroke

Baseline factors associated with global cognition in the basic model were ethnic and racial groups, study entry period, diabetes, hypertension, CVD, high cholesterol, systolic blood pressure, APOE4, depression, physical activity, and alcohol use (eTable 11 in Supplement 1). Multivariable regression revealed no evidence of multicollinearity, with variance inflation factors less than 3.2 for all covariates. The percentage of missing data was less than 6% for most covariates, except for between 9% and 38% for systolic blood pressure, high cholesterol, APOE4, and physical activity (eTable 12 in Supplement 1).

**Figure 1.**
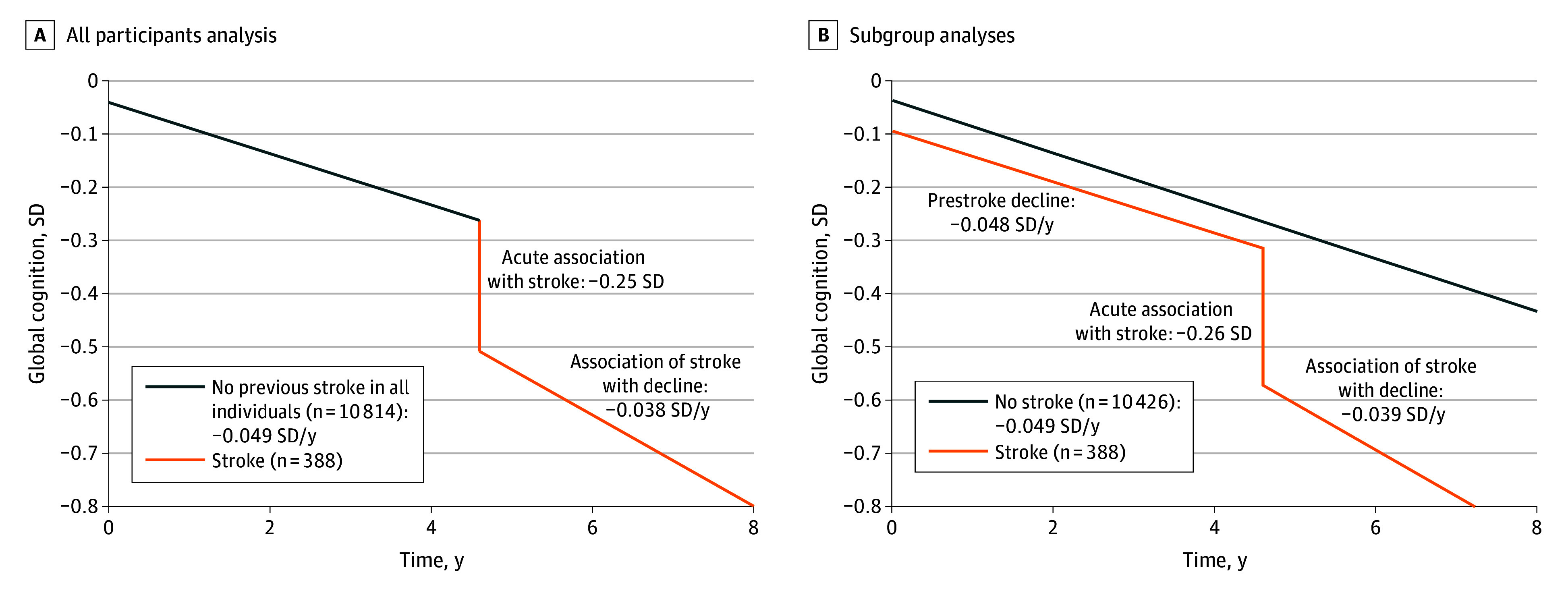
Projected Values of Global Cognition Among All Participants and in the Stroke and No-Stroke Groups Projected values of global cognition were calculated for common values of covariates at baseline and for stroke occurring at 4.6 years into the study. Common values were based on subsample with global cognition data (see eTable 12 in Supplement 1). Plots of projected values with 95% CIs are shown in eFigure 4 in Supplement 1.

Results from the adjusted model with all participants showed a poststroke acute decline of −0.251 SD (95% CI, −0.332 to −0.170 SD) in global cognition and a difference in slope of −0.038 SD per year (95% CI, −0.057 to −0.019 SD). The slope for the period without a previous stroke in all individuals was −0.049 SD per year (95% CI,−0.051 to −0.047 SD). Overall, the total poststroke slope was −0.088 SD per year (95% CI,−0.11 to −0.069 SD). Results from the unadjusted and adjusted models were similar, differing by less than 8% in effect sizes (eTable 13 in Supplement 1). Quadratic terms for TIS and TSS were not significant and were excluded. Figure 1A illustrates the results based on projected values and mean covariate values (eTable 14 in Supplement 1) for an incident stroke occurring 4.6 years (mean time to incident stroke) into the study.

#### Subgroup Analyses

The rate of change before stroke in the stroke group was −0.048 SD per year (95% CI, −0.063 to −0.033 SD) in global cognition, similar to the slope in the no-stroke group (−0.049 SD per year; 95% CI, −0.051 to −0.047 SD) (Table 3), as shown in Figure 1B. Model coefficients for stroke and TSS from the subgroup analysis were similar to those from the analysis with all participants, differing by only 3% to 4%. By including a stroke group interaction term in the model using the full sample, we showed that the slopes and baseline levels were not significantly different between the stroke and no-stroke groups (eTable 15 in Supplement 1).

**Table 3.** Adjusted Estimates of Cognitive Changes in Global Cognition Among All Participants and in the Stroke and No-Stroke Groups^a^

Measure (model variable)^c^	All participants (N = 10 814)^b^		Stroke only (n = 388)		No-stroke only (n = 10 426)	
	Coefficient (95% CI)	*P* value	Coefficient (95% CI)	*P* value	Coefficient (95% CI)	*P* value
Slope without incident stroke (TIS; SD/y)	−0.049 (−0.051 to −0.047)	<.001	−0.048 (−0.063 to −0.033)	<.001	−0.049 (−0.051 to −0.047)	<.001
Acute effect of stroke on cognitive level (stroke; SD)	−0.251 (−0.332 to −0.170)	<.001	−0.261 (−0.367 to −0.156)	<.001	NA	NA
Difference in poststroke slope relative to TIS (TSS; SD/y)	−0.038 (−0.057 to −0.019)	<.001	−0.039 (−0.064 to −0.013)	.003	NA	NA

Abbreviations: NA, not applicable; TIS, time in study; TSS, time since stroke.

^a^ The adjusted model adjusted for baseline age, sex, education, ethnic and racial groups, study entry period (before vs after 2000), history of diabetes, hypertension, cardiovascular disease, high cholesterol, systolic blood pressure, apolipoprotein E ε4 allele carrier, depression, physical activity (moderate or vigorous vs minimal activity), and alcohol use (≥1 vs <1 drink per week). Unadjusted results are shown in eTable 13 in Supplement 1.

^b^ All participants (with global cognition data) were included in the estimate of the slope without incident stroke; poststroke trajectory was estimated in those with an incident stroke (388 participants).

^c^ Additional interpretation of model coefficients: TIS = rate of decline over stroke-free trajectory; stroke = difference in intercepts between stroke-free and poststroke trajectories when TSS = 0; TSS = effect of stroke on rate of decline.

#### Sensitivity Analyses

The analysis using complete data (75% of full sample) showed slightly larger effect sizes (by 21%-24%), while excluding assessments less than 1 year after stroke resulted in no change (eTable 16 in Supplement 1). Excluding 3 studies with more than 50% loss to follow-up resulted in 66% greater poststroke difference in slope (eTable 16 in Supplement 1).

### Trajectory of Cognitive Function Poststroke in Different Domains and MMSE

The acute outcome of stroke on cognitive function was significant across all domains, with effect sizes ranging from −0.17 to −0.22 SD, as well as for the MMSE (−0.36 SD) (eTable 17 in Supplement 1). Long-term outcomes of stroke on slope were significant for language, processing speed, and executive function but not memory or the MMSE. The difference in slope after stroke was largest for processing speed (−0.055 SD per year; 95% CI, −0.076 to −0.035 SD per year) and smallest for language (−0.020 SD per year; 95% CI, −0.039 to −0.001 SD per year).

**Figure 2.**
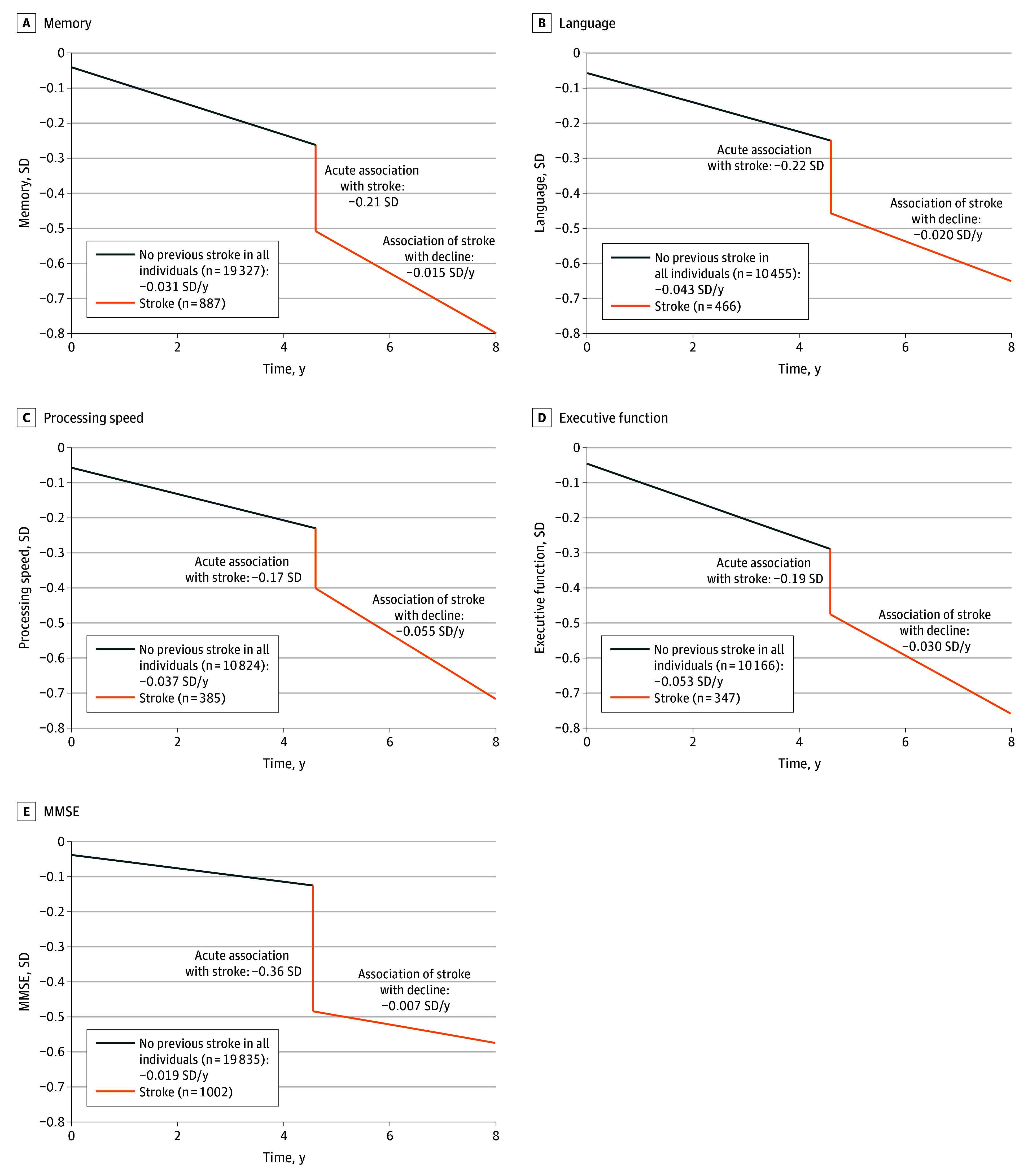
Projected Values of Cognitive Function in Each Domain and Mini-Mental State Examination (MMSE) Among All Participants Projected values of cognition scores were calculated for common values of covariates at baseline and for stroke occurring at 4.6 years into the study. Common values were based on subsample with global cognition data (see eTable 14 in Supplement 1). Plots of predicted values with 95% CIs are shown in eFigure 5 in Supplement 1.

There was no significant difference in cognitive level or slope between the stroke group before stroke and the no-stroke group in all cognitive outcomes (eTable 15 in Supplement 1). As subgroup analyses results were consistent with the full sample analysis (eTable 18 in Supplement 1), we plotted cognitive trajectories using the latter (Figure 2).

### Factors Associated With Change in Poststroke Cognitive Trajectory

Moderating effects of age, sex, education, APOE4, and vascular risk factors were investigated. Ethnic and racial groups were not examined because groups other than White (78.7%) were not well represented. None of the interaction terms were significant, except for age and acute outcome (0.013 SD; 95% CI, 0.002 to 0.023 SD) (eTable 19 in Supplement 1). To facilitate interpretation, we conducted a stratified analysis after dichotomizing age using the median value (<72 or ≥72) (eTable 20 in Supplement 1). We found that older stroke survivors experienced less acute decline, but exhibited lower cognitive levels at baseline and significantly faster decline without stroke (−0.063 SD per year vs −0.034 SD per year). eFigure 2 in Supplement 1 shows no crossing of the trajectory lines.

An interaction was found between diabetes and acute change, although it was not statistically significant (0.17 SD; 95% CI, −0.02 to 0.34 SD; *P* = .05), therefore prompting further investigation. Subgroup analyses suggested that individuals with diabetes exhibited lower cognition scores at baseline and faster, although they did not have a statistically significant decline poststroke (eTable 19 and eTable 21 in Supplement 1). Cognitive performance remained worse for those with diabetes throughout the follow-up (eFigure 3 in Supplement 1).

Although this analysis focused on examining the factors associated with cognitive change after stroke, we observed significant interactions between TIS and all vascular risk factors. This means individuals without a previous stroke who had diabetes, hypertension, CVD, high cholesterol, smoked, or carried APOE4 exhibited faster cognitive decline (eTable 19 in Supplement 1).

## Discussion

This global collaborative study involving diverse population cohorts of older adults highlights the significant and lasting negative outcomes of stroke on cognition. Incident stroke was associated with acute decline in all cognitive measures, as well as accelerated poststroke decline in global cognition, language, processing speed, and executive functioning. The prestroke cognitive trajectory of stroke survivors did not differ significantly from those without an incident stroke. There were no moderating effects of demographics or vascular risk factors on the change in cognitive trajectory after stroke, except for age.

Our results are consistent with findings from 2 previous studies.^4,5^ Others found no acceleration of decline after stroke,^6,33^ but short follow-up durations and few assessment time points may have influenced their findings. Our prior pooled analysis of 9 stroke cohorts from the Stroke and Cognition Consortium showed a poststroke decline of −0.053 SD per year in global cognition.^32^ Here, we additionally showed that decline in global cognition was faster compared with before and/or without stroke by 0.038 SD per year, and that global cognition dropped by a quarter SD after stroke, consistent across all cognitive measures (−0.17 to −0.22 SD) and similar to previous studies.^5,6^ While a change of −0.038 SD over 1 year appears small, the cumulative effect was more substantial. The combined acute and long-term effect of stroke on cognition was 0.288 SD after 1 year poststroke, equivalent to 6 years of cognitive aging in individuals without stroke, representing an important public health problem.^34^ Overall, the total decline in global cognition was 0.51 SD in just 3 years poststroke and may be considered clinically important.^4,35^

Due to varying follow-up lengths in our cohorts and the potential for missed future strokes, the estimated decline for all participants without a previous stroke of −0.049 SD per year in global cognition is particularly relevant. We also found no significant difference in prestroke cognitive trajectories compared with trajectories in those without stroke. However, potential missed stroke cases in the no-stroke group may reduce the observed difference. Our results contrast with 2 previous studies that reported faster prestroke decline compared with those without stroke.^5,7^ However, the magnitude of change was not described in 1 study,^7^ and participants were a decade younger in the other.^5^

It has been hypothesized that individuals with future stroke accumulate intracerebral damage such as cerebral small vessel disease, inflammation, and neurodegeneration via long-term exposure to vascular risk factors.^36^ However, the amount of damage sustained and extent to which these manifest as cognitive decline before stroke remain unclear. In this study, stroke survivors had higher proportions of baseline vascular risk factors compared with those without incident stroke. However, the differences were small, and adjusting for them in the models did not change the results. The older age of our participants means they may have accumulated substantial subclinical vascular brain pathology, potentially explaining the lack of significant differences in cognitive trajectories between stroke groups. The elderly may also have a higher prevalence of silent strokes or brain infarctions.^37^

Stroke may cause accelerated decline since stroke survivors have increased risk of recurrent strokes and other vascular events due to ongoing vascular damage and underlying conditions that led to the first stroke.^38^ This ongoing damage can accelerate cerebrovascular disease, promoting further brain damage, inflammation, and neurological deficits.^4,34^ Additionally, stroke-related disabilities, reduced physical and cognitive activity, and higher rates of anxiety and depression can also exacerbate cognitive decline.

In terms of cognitive domains, memory showed the smallest change in the rate of decline poststroke (−0.047 SD per year), while processing speed and executive functioning exhibited the fastest (−0.055 SD per year and −0.030 SD per year, respectively). These results support the notion of a preponderance of disturbance in processing speed and executive function among stroke survivors.^39^

The unexpected finding that older stroke survivors showed less acute decline than younger survivors may be partially explained by the older group having lower baseline cognitive scores and a possible floor effect in cognitive testing. Older stroke survivors also experienced steeper prestroke and poststroke declines, and their overall level of cognition over follow-up was worse. This finding is consistent with older adults being more likely to have neurodegenerative diseases and greater accumulation of brain pathology.^40,41^ Older adults are also more prone to severe or recurrent strokes, which are associated with faster cognitive decline and higher risk of dementia,^32,33,42^ although we lacked the data or numbers to examine this.

We did not find any vascular risk factors moderating poststroke cognitive decline, consistent with prior research.^32,43^ However, individuals without stroke, regardless of any future stroke, who had a history of diabetes, hypertension, high cholesterol, CVD, depression, smoked, or were APOE4 carriers, exhibited significantly faster cognitive decline. This supports the hypothesis that vascular risk factors exert their greatest impact on cognitive function years before stroke onset.^32^

### Strengths and Limitations

The strengths of our study include the use of diverse international cohorts, adjustments of potential confounding vascular risk factors, use of standardized scores facilitating the comparison of effect sizes, and assessments of multiple cognitive domains before and after stroke. Limitations include potential recall bias from self-reported strokes, different follow-up durations across cohorts, and lack of data on stroke characteristics. Strokes could be missed, silent, or misdiagnosed, potentially underestimating the true effects. Unmeasured confounding variables including medication use and stroke treatment could also bias our results. Since future strokes were unaccounted for in studies with shorter durations, the difference in cognitive decline before stroke compared with those without stroke may be underestimated. High attrition rates, common in longitudinal studies of older adults, resulted in older, more ill, and cognitively poorer participants dropping out. Sensitivity analyses excluding studies with high attrition rates suggested a potential underestimation of true effects due to attrition bias. Furthermore, variation in test discriminability across performance levels and differential item functioning (DIF) in cognitive testing could bias our estimation of poststroke trajectories. For example, DIF may have been present due to stroke affecting perceptual-motor abilities, resulting in lower test scores that underestimated true cognitive abilities.

## Conclusions

In this cohort study that included 14 international cohorts, we found that incident stroke was associated with substantial acute and accelerated long-term cognitive decline in older stroke survivors. Our findings could help clinicians better understand the short and long-term needs of patients with stroke. Targeting modifiable vascular risk factors at an early stage may reduce the risk of stroke but also subsequent risk of stroke-related cognitive decline and cognitive impairment. Future research should explore how modifying risk factors in midlife or later life could alter cognitive trajectories in individuals with or without incident stroke.

## Notes

Supplement 1. **eMethods. **Multiple Imputation **eTable 1. **Dementia Diagnosis Criteria and Information About Each Included Study **eTable 2. **Stroke Data From Each Study **eTable 3.** Criteria for Harmonized Baseline Factors **eTable 4. **Harmonization of Baseline Vascular Risk Factors for Each Study **eTable 5. **Missing Data for Each Variable **eTable 6. **Neuropsychological Tests Used in Each Study for Each Cognitive Domain **eTable 7. **Interpretation of Model Coefficients **eTable 8. **Participant Baseline Characteristics by Study **eTable 9. **Participant Medical History at Baseline Assessment by Study **eTable 10. **Baseline Characteristics of Participants Followed up Until the Last Assessment Versus Participants Who Dropped Out **eTable 11. **Examination of Demographic and Vascular Risk Factor Individually in the Unadjusted Model **eTable 12. **Missing Data on Covariates Included in the Adjusted Model **eTable 13. **Unadjusted Estimates of Cognitive Changes in Global Cognition **eTable 14. **Mean Values of Covariates Included the Adjusted Model **eTable 15. **Examination of Difference in Cognitive Trajectories Before Stroke Compared With Cognitive Trajectories in Those Without Stroke **eTable 16. **Sensitivity Analyses **eTable 17. **Adjusted Estimates of Changes in Cognitive Function in the 4 Cognitive Domains and MMSE Among all Participants **eTable 18.** Subgroup Analyses in Stroke Group and No-Stroke Group in Cognitive Domains and MMSE **eTable 19. **Examination of Moderating Effects in the Trajectory of Poststroke Cognitive Function **eTable 20. **Stratified Analyses for Age Groups With Global Cognition as the Outcome **eTable 21. **Stratified Analyses for Diabetes Groups With Global Cognition as the Outcome **eFigure 1. **Follow-up Schedule for Each Contributing Study **eFigure 2. **Predicted Values of Global Cognition for Participants Aged Younger Than 72 and 72 Years and Older **eFigure 3. **Predicted Values of Global Cognition for Participants With and Without Diabetes **eFigure 4. **Predicted Values of Global Cognition Among all Participants and in Subgroups, With 95% CI **eFigure 5. **Predicted Values of Cognitive Function in Each Domain and MMSE Among all Participants, With 95% CI

Supplement 2.
Data Sharing Statement
