# Supplementary material for: Trajectory of Cognitive Decline Before and After Stroke in 14 Population Cohorts
Source: JAMA Netw Open. 2024 Oct 2;7(10):e2437133. doi: 10.1001/jamanetworkopen.2024.37133 (PMC11447567; doi:10.1001/jamanetworkopen.2024.37133)
Supplement: Supplement 1. — eMethods. Multiple Imputation eTable 1. Dementia Diagnosis Criteria and Information About Each Included Study eTable 2. Stroke Data From Each Study eTable 3. Criteria for Harmonized Baseline Factors eTable 4. Harmonization of Baseline Vascular Risk Factors for Each Study eTable 5. Missing Data for Each Variable eTable 6. Neuropsychological Tests Used in Each Study for Each Cognitive Domain eTable 7. Interpretation of Model Coefficients eTable 8. Participant Baseline Characteristics by Study eTable 9. Participant Medical History at Baseline Assessment by Study eTable 10. Baseline Characteristics of Participants Followed up Until the Last Assessment Versus Participants Who Dropped Out eTable 11. Examination of Demographic and Vascular Risk Factor Individually in the Unadjusted Model eTable 12. Missing Data on Covariates Included in the Adjusted Model eTable 13. Unadjusted Estimates of Cognitive Changes in Global Cognition eTable 14. Mean Values of Covariates Included the Adjusted Model eTable 15. Examination of Difference in Cognitive Trajectories Before Stroke Compared With Cognitive Trajectories in Those Without Stroke eTable 16. Sensitivity Analyses eTable 17. Adjusted Estimates of Changes in Cognitive Function in the 4 Cognitive Domains and MMSE Among all Participants eTable 18. Subgroup Analyses in Stroke Group and No-Stroke Group in Cognitive Domains and MMSE eTable 19. Examination of Moderating Effects in the Trajectory of Poststroke Cognitive Function eTable 20. Stratified Analyses for Age Groups With Global Cognition as the Outcome eTable 21. Stratified Analyses for Diabetes Groups With Global Cognition as the Outcome eFigure 1. Follow-up Schedule for Each Contributing Study eFigure 2. Predicted Values of Global Cognition for Participants Aged Younger Than 72 and 72 Years and Older eFigure 3. Predicted Values of Global Cognition for Participants With and Without Diabetes eFigure 4. Predicted Values of Global Cognition Among all Participants and in Subgroups, [file jamanetwopen-e2437133-s001.pdf]

Supplemental Online Content

Lo JW, Crawford JD, Lipnicki DM, et al. Trajectory of cognitive decline before and after stroke in 14 population cohorts. *JAMA Netw Open*. 2024;7(10):e2437133. doi:10.1001/jamanetworkopen.2024.37133

eMethods. Multiple Imputation

eTable 1. Dementia Diagnosis Criteria and Information About Each Included Study

eTable 2. Stroke Data From Each Study

eTable 3. Criteria for Harmonized Baseline Factors

eTable 4. Harmonization of Baseline Vascular Risk Factors for Each Study

eTable 5. Missing Data for Each Variable

eTable 6. Neuropsychological Tests Used in Each Study for Each Cognitive Domain

eTable 7. Interpretation of Model Coefficients

eTable 8. Participant Baseline Characteristics by Study

eTable 9. Participant Medical History at Baseline Assessment by Study

eTable 10. Baseline Characteristics of Participants Followed up Until the Last Assessment Versus Participants Who Dropped Out

eTable 11. Examination of Demographic and Vascular Risk Factor Individually in the Unadjusted Model

eTable 12. Missing Data on Covariates Included in the Adjusted Model

eTable 13. Unadjusted Estimates of Cognitive Changes in Global Cognition

eTable 14. Mean Values of Covariates Included the Adjusted Model

eTable 15. Examination of Difference in Cognitive Trajectories Before Stroke Compared With Cognitive Trajectories in Those Without Stroke

eTable 16. Sensitivity Analyses

eTable 17. Adjusted Estimates of Changes in Cognitive Function in the 4 Cognitive Domains and MMSE Among all Participants

eTable 18. Subgroup Analyses in Stroke Group and No-Stroke Group in Cognitive Domains and MMSE

eTable 19. Examination of Moderating Effects in the Trajectory of Poststroke Cognitive Function

© 2024 Lo JW et al. *JAMA Network Open.*

eTable 20. Stratified Analyses for Age Groups With Global Cognition as the Outcome

eTable 21. Stratified Analyses for Diabetes Groups With Global Cognition as the Outcome

eFigure 1. Follow-up Schedule for Each Contributing Study

eFigure 2. Predicted Values of Global Cognition for Participants Aged Younger Than 72 and 72 Years and Older

eFigure 3. Predicted Values of Global Cognition for Participants With and Without Diabetes

eFigure 4. Predicted Values of Global Cognition Among all Participants and in Subgroups, With 95% CI

eFigure 5. Predicted Values of Cognitive Function in Each Domain and MMSE Among all Participants, With 95% CI

This supplemental material has been provided by the authors to give readers additional information about their work.

© 2024 Lo JW et al. *JAMA Network Open.*

eMethods. Multiple Imputation

We assumed that missing data was missing at random (MAR) and multiple imputation (MI) using chained equations was used to impute missing covariates. All baseline covariates including baseline global cognition and domain scores, as well as demographic data were included in the imputation model. Follow up data were not included in the imputation because each study and participant have different follow up schedules. Since some covariates (APOE4, CVD, high cholesterol, and physical activity) were missing completely in some studies, imputation was performed in the full sample. Since the clustering of studies was not the focus of our analysis, we ignored the clustering of studies in the imputation model for simplicity and only allow for it in the analysis model [White et al. 2011].

The fraction of incomplete data in the adjusted model was about 20 per cent, hence we used 20 imputations as recommended in [White et al. 2011].

Reference:

White IR, Royston P, Wood AM. Multiple imputation using chained equations: Issues and guidance for practice. Stat Med. 2011;30(4):377-99. doi: 10.1002/sim.4067.

© 2024 Lo JW et al. *JAMA Network Open.*

eTable 1. Dementia Diagnosis Criteria and Information About Each Included Study

Study name (acronym)	Diagnostic criteria for dementia at baseline [a]	About study/ Recruitment strategies [b]
Einstein Aging Study (**EAS**)	DSM-IV	Between 1993 and 2004, Health Care Financing Administration/ Centers for Medicaid and Medicare Services (HCFA/CMS) rosters of Medicare eligible persons aged 70 and above were used to develop sampling frames of community residing participants in Bronx County in the US. Since 2004, New York City Board of Elections registered voter lists for the Bronx have been used due to changes in policies for release of HCFA/CMS rosters.
Epidemiology of Dementia in Central Africa (**EPIDEMCA**)	DSM-IV and NINCDS-ADRDA criteria	A multicenter population-based study was carried out in Central African Republic and Republic of Congo between 2011 and 2012 including both urban and rural sites in each country. Follow up was performed in Congo only and therefore, the sample for this project included the subsample from Congo.
EpiFloripa Aging Study (**EpiFloripa)**	MMSE (≤ 17 [c])	The EpiFloripa Aging Study was carried out in the urban area of Florianópolis, State of Santa Catarina, located in the southern region of Brazil. The sample selection process was carried out by conglomerates in two steps. The units of the first step were the census tracts (IBGE census units) and the units of the second step were the households. Households were randomly selected. Mortality data linkage and active search for participants were used as follow-up strategies.
Etude SanteÂ Psychologique et Traitement (**ESPRIT**)	Neurologist interviews with cognitive testing; diagnoses validated by an independent expert panel	The Esprit Study is a neuropsychiatric cohort study of community dwelling people aged 65 years and over drawn at random from the electoral rolls of Montpellier in the south of France and recruited between 1999 and 2001.
Gothenburg H70 Birth Cohort Studies (**H70 study**)	DSM-III-R	The Gothenburg H70 Birth Cohort Studies are multidisciplinary epidemiological studies examining representative birth cohorts of older populations in Gothenburg, Sweden. The first study started in 1971. Each birth cohorts were systematically selected from the Swedish Revenue Office Register. Participants could opt to take the examination over several days, and home visits were also offered as an alternative.
Invecchiamento Cerebrale in Abbiategrasso (**Invece.Ab**)	DSM-IV	Eligible population comprises all people born between 1935 and 1939 who were residents living in Abbiategrasso, Milan, Italy on the start date of the study. All 1773 people born between 1935–39 residing in Abbiategrasso were contacted, a list obtained from the municipal registry office. Personalization of successive contacts were used.
Leipzig Longitudinal Study of the Aged (**LEILA75+**)	DSM-IV	A total of 1500 community-dwelling individuals aged 75 years and over living in the community of Leipzig-South were identified by systematic random sampling from an age-ordered list provided by the official registry office for each of the seven subdistricts. Face-to-face interviews were conducted, and proxy interviews were performed with relatives of fragile and functionally dependent individuals.
**Study name (acronym)**	**Diagnostic criteria for dementia at baseline [a]**	**About study/ Recruitment strategies [b]**

© 2024 Lo JW et al. *JAMA Network Open.*

Monongahela-Youghiogheny Healthy Aging Team (**MYHAT**)	CDR (≥ 1)	An age-stratified sample of 2036 individuals aged 65+ years was drawn from the electoral rolls of a U.S. community, excluding individuals with severe cognitive impairment.
Personality and Total Health Through Life Project (**PATH**)	DSM-IV	The sample was drawn from the electoral rolls of the three federal electorates that make up the Australian Capital Territory and the electorate containing Queanbeyan. From this latter electorate, we selected only those giving Queanbeyan as their residential address. To assist follow-up, participants were asked to provide the name, address, and phone numbers of two contacts (friends or relatives). Participants were sent a card, a PATH Newsletter and a 'change of address' card. Participants were asked to update the information on these cards and post them back or telephone or email with changes to their contact details.
Sacramento Area Latino Study on Aging (**SALSA**)	California ADDTC criteria and NINDS-ADRDA	The SALSA Study was a longitudinal cohort study of 1,789 community-dwelling Mexican Americans residing in California's Sacramento Valley who were aged 60-101 years at baseline in 1998-1999. Participants were followed every 12-15 months via home visits that included clinical and cognitive assessments. A semi-annual phone call was made to obtain updates on medications, health events, and some sociodemographic risk factors.
Sydney Memory and Ageing Study (**Sydney MAS**)	DSM-IV	Non-demented community-dwelling individuals aged 70–90 were recruited from two areas of Sydney, following a random approach to 8914 individuals on the electoral roll.
Taiwan Initiative for Geriatric Epidemiological Research (**TIGER**)	A history of dementia – self-report or medication use and MOCA-T (≤ 19) [d]	Adults aged ≥ 65 years who participated in the senior health checkup program at National Taiwan University Hospital during 2011–2013 were recruited.
Vallecas Project (**Vallecas**)	DSM-IV-TR (text-revised)	Volunteers were recruited through radio and TV campaigns, leaflet distribution, and visits of the research team to social centers for the elderly.<br>If a participant cannot attend a follow-up visit, he/she is invited to perform a medical interview by phone.
Zaragoza Dementia Depression Project (**ZARADEMP**)	DSM-IV	Random sample from the census list in the city of Zaragoza (1991) of both, men and women aged 55 years or more, stratified by sex and age (5-year age categories). To minimize attrition, a proportion of the elderly were traced to their children's homes or to their temporary rural homes.

[a] Participants with dementia at baseline were excluded from the analyses.
[b] For further details about each study, refer to the reference paper listed in Table 1.
[c] Dementia was not diagnosed in the study. The lowest 5th percentile (= cut-off point of 17) is used to determine dementia, as recommended for the use in Brazilian population-based studies of elderly with low schooling level [Castro-Costa 2008].
[d] MoCA-T = Montreal Cognitive Assessment-Taiwanese version. Dementia was not diagnosed in this study. There is no established cut-off for dementia for the MoCA-T and we excluded those in the lowest 5th percentile (= cut-off point of 19) which is a conservative estimate for suspected dementia, as a large survey in Taiwan reported age-gender-adjusted prevalence of 8.1% for all cause dementia in adults aged 65+ [Sun 2014].

References:
Castro-Costa E, Fuzikawa C, Uchoa E, Firmo JO, Lima-Costa MF. Norms for the mini-mental state examination: adjustment of the cut-off point in population-based studies. Arq Neuropsiquiatr. 2008;66:524-8. doi: 10.1590/s0004-282x2008000400016.
Sun Y, Lee HJ, Yang SC, et al. A nationwide survey of mild cognitive impairment and dementia, including very mild dementia, in Taiwan. PLoS One. 2014;9(6):e100303. doi: 10.1371/journal.pone.0100303.

© 2024 Lo JW et al. *JAMA Network Open.*

eTable 2. Stroke Data From Each Study

Study [a]	Definition of incident stroke	Time of stroke (since study entry)
**EAS**	Self-reported, occurred in the past year	Time in study for the wave (which recorded the stroke) minus 0.5
**EPIDEMCA**	Self-reported: Have you ever had an attack which requested medical attention? Or requiring hospitalization or consult due to stroke in the last 12 months	Time in study for the wave (which recorded the stroke) minus 0.5
**Epifloripa**	Self-reported: Has any doctor or health professional ever said that you have stroke	Halfway between two waves
**ESPRIT**	Self-reported	Time in study for the wave (which recorded the stroke) minus 1 year (for wave 4, minus 1.5)
**H70 study**	History of stroke, based on in-patient register and/or from examination	Year of stroke
**Invece.Ab**	World Health Organization (WHO) definition of stroke or TIA used (Note, TIAs were not distinguished from stroke)	Year and month of stroke
**LEILA 75+**	Self-reported	Halfway between two waves
**MYHAT**	Self-reported	Halfway between two waves
**PATH**	Self-reported (any stroke since the last assessment)	Halfway between two waves or based the self-reported year which stroke occurred (1st June of the year)
**SALSA**	Self-report or stroke listed as cause of death in death cert	Self-reported dates
**Sydney MAS**	Self-reported diagnosis of a stroke in the last 2 years (for wave 5: in the past 12 months)	> 5 years ago, 1-2 years ago, 6-12 months ago, or 3-6 months ago
**TIGER**	Self-reported at wave 3 and 4 (information was not collected at wave 2)	Halfway between two waves
**Vallecas**	Self-reported	Halfway between two waves
**ZARADEMP**	Self-reported (Has a doctor told you that you had a stroke?)	Halfway between two waves

[a] For full study names refer to eTable 1.

© 2024 Lo JW et al. *JAMA Network Open.*

eTable 3. Criteria for Harmonized Baseline Factors

Baseline factor	Categories [a]	Criteria (meeting any is sufficient)
Diabetes	Yes/no	Medical history, treatment, self-report, and/or fasting blood glucose ≥ 126mg/dL or > 7mmol/L.
Hypertension	Yes/no	Medical history, treatment, self-report, and/or seated systolic blood pressure ≥ 140 mmHg or diastolic blood pressure ≥ 90 mmHg.
High cholesterol	Yes/no	Medical history, treatment, self-report, and/or total cholesterol ≥ 240mg/dL (> 6.2mmol/L), or triglycerides ≥ 200mg/dL (> 2.3mmol/L), or LDL ≥ 160 mg/dL (> 4.1 mmol/L).
Cardiovascular disease	Yes/no	Atrial fibrillation, coronary heart disease, angina, myocardial infraction, heart failure, and/or ischemic heart disease self-reported or in the medical history notes.
Depression	Yes/no	Use of anti-depressants, self-reported depression, and/or depression based on a depression scale [b] and associated cut-off point as noted for individual study.
Smoking	Never, past, current [b]	Self-reported.
Alcohol use	No/minimal, 1 drink/week, ≥ 2 drinks/week [b]	Based on estimated/self-reported number of drinks per week.
Physical activity	Minimal, moderate, vigorous [b]	Based on physical activity questionnaires [b] where different types of activities such as walking, gardening, swimming, etc. and time spent on the activities per week were recorded.

[a] Harmonized categories used in the present paper.
[b] For the key model in the statistical analysis, the 3 categories were collapsed into two (no/minimal vs the other categories) to allow convergence in the multiple imputation of the covariates.

© 2024 Lo JW et al. *JAMA Network Open.*

eTable 4. Harmonization of Baseline Vascular Risk Factors for Each Study

Study [a]	Criteria (meeting any is sufficient)					
	**Diabetes**	**Hypertension**	**High cholesterol**	**Cardiovascular disease**	**Alcohol use**	**Depression**
**EAS**	Medical history	Blood pressure (mean of 2), medical history	Cholesterol, triglycerides	Myocardial infarction, coronary artery bypass, angina, heart failure, angioplasty, or arrhythmia	Drinks per week calculated from use during past year for each of beer, wine, liquor	GDS score 6+
**EPIDEMCA**	Medical history, treatment, blood glucose	Blood pressure (mean of 2), medical history, treatment	Cholesterol [c]	NA	Number of alcohol unit in a 'normal' week	GMS-AGECAT rating of subcase or clinical case
**Epifloripa**	Self-report, fasting blood glucose [b]	Blood pressure (mean of 2), self-report	Cholesterol, triglycerides, LDL	Self-report	According to the AUDIT Alcohol screening tool. Never=0; Moderate=1; High=2	Depression observed during neuropsychiatric exam
**ESPRIT**	Treatment, fasting blood glucose	Blood pressure (mean of 2), medication	Treatment, cholesterol, triglycerides	Ischemic heart disease (angina, history of angioplasty, heart operation or myocardial infarction), heartbeat disorders (arrhythmia or auricular fibrillation)	Drinks per week calculated from consumption in grams/day using 10 g = 1 drink	GDS score 6+; self-reported depression
**H70 study**	Self-report, medication	Self-reported with treatment, blood pressure	Dyslipidemia [d]	Myocardial infarction, angina, heart failure, or atrial fibrillation (self-report)	Estimated total alcohol consumption per week in g/week.	Major (according to DSM-V criteria) or minor (according to DSM-IV-TR criteria) depression; use of anti-depressants
**Invece.Ab**	Treatment, medical history	Blood pressure, medication	Medical history, treatment	Myocardial infarction, heart failure, angina, arrhythmia, coronary artery bypass graft, atrial fibrillation (medical history)	Whether an individual drinks alcohol habitually, regardless of the amount. In Italy it is part of the normal diet to drink wine with meals. Data coded 0 or 1 only.	Use of anti-depressants; GDS score 6+; Criteria-based diagnosis by physician/ psychologist (including medication, GDS score and CES-D items)

© 2024 Lo JW et al. *JAMA Network Open.*

Study [a]	Criteria (meeting any is sufficient)					
	**Diabetes**	**Hypertension**	**High cholesterol**	**Cardiovascular disease**	**Alcohol use**	**Depression**
**LEILA 75+**	Self-reported	Blood pressure, medication	NA	Myocardial infarction (self-report)	Number of drinks per week	DSM-IV criteria based on structured clinical interview; CES-D score 16+
**MYHAT**	Self-reported	Medical history, medication, blood pressure (average of measures from waves 1 and 2)	Self-reported	Myocardial infarction, coronary heart failure	Calculated based on questions "Have you ever had alcohol?", "How often do you drink?", "How many drinks do you have at a time?"	Use of anti-depressants; CES-D (modified) score ≥ 3
**PATH**	Medical history, treatment	Blood pressure (mean of 2), medication	Treatment	“Do you have heart trouble?”	Drinks per week coded as monthly or less = 0, 2-4 times a month = 1, 2-3 times a week or more = 2	Goldberg Anxiety and Depression Scale depression score 6+; Anti-depressants taken
**SALSA**	Self-report, fasting blood glucose, medication	Blood pressure (mean of 2), medication	Medication, cholesterol, triglycerides	Myocardial infarction, angina, congestive heart failure, atrial fibrillation, heart catheterization (self-report)	Number of drinks per week	CES-D score 16+; Anti-depressants taken
**Sydney MAS**	Fasting blood glucose, treatment, medical history	Blood pressure (mean of 2), self-report, medication	Medical history, treatment, cholesterol, triglycerides	Heart attack, angina, cardiomyopathy, valve disease, arrhythmia, atrial fibrillation (ever diagnosed)	Coded as monthly or less = 0, 2-4 times a month = 1, 2-3 times a week or more = 2	GDS score 6+; use of medication
**TIGER**	Self-report, medication, fasting blood glucose	Self-report, medication	Cholesterol, self-report (for hyperlipidemia), medication	Coronary heart disease, atrial fibrillation (self-report)	Never or ever	Self-report; Medication use; CES-D score ≥ 16
**Vallecas**	Fasting blood glucose, treatment (not including diet)	Self-report, medication	Self-report (high cholesterol or high triglycerides)	Angina or myocardial infarction	Converted from grams of alcohol p/week* to drinks p/week (divide by 14g); < 1 = 0; > 0 and < 2 drink = 1; 2 or more = 2	Diagnosed by a physician (including medication and treatment); GDS score 6+

© 2024 Lo JW et al. *JAMA Network Open.*

Study [a]	Criteria (meeting any is sufficient)					
	**Diabetes**	**Hypertension**	**High cholesterol**	**Cardiovascular disease**	**Alcohol use**	**Depression**
**ZARADEMP**	Medical history, treatment	Blood pressure (mean of 2), medication	Self-report (dyslipidemia)	Myocardial infarction, angina (diagnosed using EURODEM Risk Factor Questionnaire and medical records)	Yes or no	GMS-AGECAT rating of subcase or clinical case

All medical history data were collected at baseline unless specified.
Abbreviations: NA=not available. CES-D, Centre for Epidemiological Studies depression scale. CIRS, Cumulative Illness Rating Scale. DSM-IV, Diagnostic and Statistical Manual of Mental Disorders (4th edition). GDS, Geriatric Depression Scale. GMS-AGECAT, Geriatric Mental State-Automated Geriatric Examination for Computer Assisted Taxonomy. ICD-10, International Classification of Diseases (10th revision) MINI, Mini International Neuropsychiatric Interview, NPI, Neuropsychiatric Inventory.
[a] For full study names refer to eTable 1.
[b] High cholesterol was defined as (>5.3mmol/L) for EPIDEMCA.
[c] Collected at the second wave but not at baseline.
[d] For the H70 study, the definition of dyslipidemia was total cholesterol/HDL >=5 or LDL >= 3.5 or on lipid lowering drugs.

© 2024 Lo JW et al. *JAMA Network Open.*

eTable 4. Harmonization and Definition of Baseline Vascular Risk Factors (continued from previous table)

Study	Physical activity<br>Minimally active = 0; Moderate activity at least once per week = 1; Vigorous activity at least once per week = 2
EAS	Light activities (walking, gardening, dancing, calisthenics, golf, bowling, horse riding) at least 1 day a week = 1, Medium (hiking, tennis, cycling, swimming) or heavy (jogging, aerobics, hand, or racquet ball) activities at least 1 day a week=2, 0 (or less) days for all categories=0
EPIDEMCA	Current physical activity was estimated using a thresh- old of at least 150 minutes of walking or cycling in the past week (World Health Organization 1984).
Epifloripa	The physical activity score was measured using the long version of the International Physical Activity Questionnaire, adapted, and validated for the older adults in Brazil. The recreation and transportation domains were used. 1 ≥ 150 min/week, 0 ≤ 150 min/week.[a]
ESPRIT	Either of gardening or walking “a little” or “a lot” = 1 vs “very little” = 0, Sports “regularly” or “often” = 2, gardening or walking “very little” and sports “never” or “from time to time” = 0
H70 study	0 = "almost nothing" or "mostly sedentary, walking sometimes/easy gardening/light household work". 1 = walks at least once per week; "Light physical activity, 2-4 h/w e.g. walks, fishing, dancing" or "Moderate physical activity, 1-2 h/w e.g. exercise, swimming, gymnastics, all household work". 2 = "Moderate physical activity, at least 3 times/w e.g. tennis, swimming, exercising, running" or "Intense physical activity, regularly several times/w"
Invece.Ab	Response options are “never”, “1 x week”, “2 x week”, “3+ week”. Any of walking > 30 mins, dancing, “others”, group exercise “1 x week” or more = 1. Any of cycling, swimming, running-jogging, tennis, aerobics “1 x week” or more = 2. None of these or only walking < 30 mins = 0.
LEILA75+	NA
MYHAT	Classed as bivariate
PATH	Moderate activity (scrubbing, polishing car, dancing, golf, cycling, decorating, lawn mowing, leisurely swimming) “once or twice a week” or “3 times a week or more” = 1. Vigorous activity (running, hard swimming, tennis, squash, digging, cycle racing) “once or twice a week” “3 times a week or more” = 2. Less frequent participation in these, only mild activity (walking, woodwork, gardening, bike repairs, playing pool, general housework), or less = 0.
SALSA	1 hour or more of swim or work out = 2; doing yard work, taking walks, doing heavy housework, dancing, hunting or camping or boating, golf or other moderate exercise, walking around your neighborhood, or climbing at least 5 flights of stairs per day [b] = 1; doing house repairs, baking, doing light housework, cook meals, standing or walking at work or home, sitting at work or home, driving a car.
Sydney MAS	Any time indicated for separate activities bowls, golf, dancing, walking, other (Pilates, yoga, tai chi, weights)=1. Any time indicated for tennis, swimming, jogging, bicycling, aerobics, other relevant=2. Cases with no time indicated or where the time is clearly less than once a week=0.
TIGER	A short version of International Physical Activity Questionnaire (IPAQ) [c] was used to measure physical activity in MET minutes per week.<br>≥ 1500 MET-min/week = 2; ≥ 600 and < 1500 MET-min/week = 1; < 600 MET-min/week = 0.
Vallecas	NA
ZARADEMP	NA

Note. All information provided by the investigator or data manager of each study. NA=not available. [a] Ono LM, Schneider IJ, Confortin SC, d'Orsi E. Paid Work and Physical Activity Preserve Functional Capacity in Elderly People: EpiFloripa Study. Gerontol Geriatr Med. 2015;1:2333721415608022. [b] In total energy expenditure, 5 flights of stairs per day was calculated as approximately equal to one 30-min walk a week: walking 1 block (0.13 km) daily = 235 kJ/week; climbing up and down 1 flight of stairs daily = 118 kJ/week; and assuming a moderate walking pace of 4.8 km/h. Lee IM, Paffenbarger RS, Jr. Associations of light, moderate, and vigorous intensity physical activity with longevity. The Harvard Alumni Health Study. Am J Epidemiol 2000;151:293-9.
[c] International Physical Activity Questionnaire. IPAQ scoring protocol. https://sites.google.com/view/ipaq/score [accessed 28th February 2024]

© 2024 Lo JW et al. *JAMA Network Open.*

eTable 5. Missing Data for Each Variable

Baseline factor	Available data	Missing data, %
Age at baseline	20860	0
Study entry period	20860	0
sex	20860	0
Education	20860	0
Ethno-racial groups	20860	0
BMI	18201	0
APOE ε4 carrier	13096	37.2%
Blood pressure	18904	9.4%
Diabetes	20735	0.6%
Hypertension	20746	0.5%
High cholesterol	17910	14.1%
Cardiovascular disease	19779	5.2%
Smoker (ever)	20771	0.4%
Alcohol use	19675	5.7%
Physical activity	13009	37.6%
Depression	19755	5.3%
**Baseline cognitive scores**		
MMSE	19829	4.9%
Global cognition	8483	59.3%
Processing speed	8554	59.0%
Memory	19163	8.1%
Language	10458	49.9%
Executive function	7815	62.5%

© 2024 Lo JW et al. *JAMA Network Open.*

eTable 6. Neuropsychological Tests Used in Each Study for Each Cognitive Domain

Study [a]	Memory	Language	Processing Speed	Executive Function	Mini-Mental State Examination (MMSE)	Global Cognition [b]
**EAS**	Free and Cued Selective Reminding Test	Animals in 60s	TMTA	TMTB	Converted from Blessed	Yes
**EPIDEMCA**	Free and Cued Selective Reminding Test	Animals/ fruits in 60s	Zazzo's Cancellation Task	NA	NA	NA
**EpiFloripa**	NA	NA	NA	NA	Yes	NA
**ESPRIT**	5-word test	Animals in 30s	TMTA	TMTB	Yes	Yes
**H70 study**	Memory in Reality test (3 waves only)	Animals	NA	NA	Yes	NA
**Invece.Ab**	Rey Auditory Verbal Learning Test, trial 7 (delay=15 min)	Verbal fluency, mean of colors/ animals/ fruits/ cities, each 120s	TMTA	TMTB	Yes	Yes
**LEILA75+**	SIDAM word list	NA	NA	NA	Yes	NA
**MYHAT**	FULD	Animals	TMTA	TMTB	Yes	Yes
**PATH**	Delayed recall (wave 4 was not included [c])	NA	TMTA (baseline NA)	TMTB (baseline NA)	Yes	Yes (baseline and wave 4 NA)
**SALSA**	Word list	NA	NA	NA	Yes	NA
**Sydney MAS**	RAVLT	Animals in 60s	TMTA	TMTB	Yes	Yes
**TIGER**	Delayed free recall	Verbal fluency, mean of (fruits/fish/ vegetables	TMTA	TMTB	NA	Yes
**Vallecas**	Delayed free recall	Animals	NA	NA	Yes	NA
**ZARADEMP**	MMSE – word list	NA	NA	NA	Yes	NA

Abbreviations: NA=not available. TMTA=Trail Making Test A; TMTB=Trail Making Test B.
[a] For full study names refer to eTable 1.
[b] Global cognition was calculated as the average of at least 3 domains.
[c] Delayed recalled scores from wave 4 were not included since a different version of the test was used.

© 2024 Lo JW et al. *JAMA Network Open.*

eTable 7. Interpretation of Model Coefficients

Analysis using:	Model interpretation of coefficient (condition)	Clinical interpretation
**1) All participants**		
TIS	Slope (stroke=0 for all participants)	Rate of decline (SD/y) without a previous stroke in all participants (“stroke-free trajectory”)
TSS	Difference in slope in relation to TIS (stroke=1)	Long-term effect of stroke on cognitive decline
Stroke	Difference in intercepts between two functions (TSS=0)	Acute effect of stroke on level of cognition
**2) Stroke group only**		
TIS	Slope before stroke (stroke=0)	Rate of decline (SD/y) before a stroke event (“pre-stroke trajectory”)
TSS	Difference in slope after stroke (stroke=1)	Long-term effect of stroke on cognitive decline (change in slope post-stroke)
Stroke	Difference in intercepts between two functions (TSS=0)	Acute effect of stroke on level of cognition (acute change post-stroke)
**3) No-stroke group only**		
TIS	Slope for no-stroke group only	Rate of decline in participants without stroke over follow up
TSS	Not included	NA
Stroke	Not included	NA

© 2024 Lo JW et al. *JAMA Network Open.*

eTable 8. Participant Baseline Characteristics by Study

Study [a]	N	Age (years)	Male	Education (years)
**EAS**	1,915	78.1 (5.3)	37%	13.2 (3.6)
**EPIDEMCA**	448	74.1 (7.1)	37%	2.21 (4.1)
**EpiFloripa**	1,054	69.2 (7.0)	34%	8.2 (5.8)
**ESPRIT**	2,098	73.0 (5.5)	41%	10.2 (3.8)
**H70 study**	550	70.2 (1.0)	41%	10.2 (4.4)
**Invece.Ab**	1,082	72.1 (1.3)	46%	7.1 (3.3)
**LEILA75+**	878	81.5 (4.8)	25%	11.9 (1.8)
**MYHAT**	1,808	77.5 (7.4)	39%	12.9 (2.4)
**PATH**	2,420	62.5 (1.5)	52%	13.8 (2.8)
**SALSA**	1,565	70.2 (6.8)	41%	7.3 (5.4)
**Sydney MAS**	996	78.9 (4.8)	47%	11.6 (3.5)
**TIGER**	566	72.9 (5.4)	37%	13.6 (3.7)
**Vallecas**	1,103	74.9 (3.9)	36%	10.3 (5.8)
**ZARADEMP**	4,377	73.2 (9.5)	43%	7.2 (3.9)
**TOTAL**	20,980	72.9 (8.0)<br>Range = 58 – 103	43%	10.0 (4.9)

Note. Figures represent mean (SD) or n (%). A representative from each study has checked their study data reported in this table.
[a] For full study names refer to eTable 1.

© 2024 Lo JW et al. *JAMA Network Open.*

eTable 9. Participant Medical History at Baseline Assessment by Study

Study [a]	N [b]	APOE4 carrier [c]	BMI	Systolic BP	Hypertension [d]	Diabetes [d]	High Cholesterol [d]	CVD [d]	Smoking (ever) [e]	Alcohol [f]	Physically active [g]	Depression [d]
**EAS**	1,915	213/968 (22%)	28.3 (5.3)	134 (16)	1165/1861 (63%)	322/1870 (17%)	95/586 (16%)	613/1878 (33%)	1024/1877 (55%)	648/1353 (48%)	472/766 (62%)	180/1570 (11%)
**EPIDEMCA**	448	126/304 (41%)	21.6 (5.1)	146 (30)	323/448 (72%)	48/441 (11%)	50/367 (14%)	NA	77/448 (17%)	72/442 (16%)	240/447 (54%)	14/125 (11%)
**EpiFloripa**	1,054	NA	28.2 (5.0)	141 (21)	808/1054 (77%)	642/1054 (61%)	118/542 (22%)	274/1054 (26%)	399/1054 (38%)	400/1054 (38%)	534/504 (51%)	157/1032 (15%)
**ESPRIT**	2,098	391/2059 (19%)	25.1 (3.7)	141 (17)	1499/2098 (71%)	193/2098 (9%)	1187/2098 (57%)	411/2098 (20%)	865/2097 (41%)	1551/2061 (75%)	1566/1866 (84%)	633/2089 (30%)
**H70 study**	550	155/539 (29%)	27.0 (4.2)	155 (22)	453/550 (82%)	51/524 (10%)	380/546 (70%)	86/550 (16%)	282/519 (54%)	119/518 (23%)	448/450 (~100%)	95/550 (17%)
**Invece.Ab**	1,082	195/1081 (18%)	27.1 (4.6)	142 (18)	641/1082 (59%)	179/1081 (17%)	352/1081 (33%)	288/1081 (27%)	453/1082 (42%)	687/1081 (64%)	742/1082 (69%)	166/1082 (15%)
**LEILA75+**	878	38/230 (17%)	NA	159 (24)	660/822 (80%)	194/878 (22%)	NA	74/878 (8%)	278/873 (32%)	768/874 (88%)	NA	291/878 (33%)
**MYHAT**	1,808	344/1643 (21%)	28.0 (5.5)	133 (15)	1485/1808 (82%)	387/1806 (21%)	1091/1800 (61%)	350/1803 (19%)	957/1804 (53%)	478/1808 (26%)	1543/1806 (85%)	384/1804 (21%)
**PATH**	2,420	615/2263 (27%)	26.9 (5.3)	140 (19)	1582/2418 (65%)	177/2418 (7%)	535/2419 (22%)	338/2414 (14%)	1153/2418 (48%)	1696/2417 (70%)	1637/2411 (68%)	217/2419 (9%)
**SALSA**	1,565	193/1417 (14%)	29.8 (6.0)	138 (19)	1033/1565 (66%)	484/1565 (31%)	726/1436 (51%)	315/1547 (20%)	838/1565 (54%)	363/1561 (23%)	1382/1565 (88%)	439/1555 (28%)
**Sydney MAS**	996	214/926 (23%)	27.1 (4.5)	145 (21)	824/996 (83%)	153/996 (15%)	675/996 (68%)	288/996 (29%)	533/994 (54%)	611/996 (61%)	812/996 (82%)	144/996 (14%)
**TIGER**	566	89/563 (16%)	23.9 (2.9)	127 (15)	346/566 (61%)	90/566 (16%)	274/566 (48%)	133/565 (24%)	91/566 (16%)	116/566 (20%)	467/566 (83%)	55/566 (10%)
**Vallecas**	1,103	186/1103	27.3 (3.6)	143 (21)	577/1102 (52%)	127/1102 (12%)	588/1096 (54%)	59/1097 (5%)	424/1102 (38%)	96/576 (17%)	NA	371/1102 (34%)
**ZARADEMP**	4,377	NA	27.0 (5.0)	141 (19)	2977/4376 (68%)	542/4336 (13%)	245/4377 (6%)	295/4368 (7%)	1512/4372 (35%)	1144/4368 (26%)	NA	764/3987 (19%)

Note. Figures represent mean (SD) or n (%). A representative from each study has checked their study data reported in this table. [a] For full study names refer to eTable 1. [b] Sample size for the present project which included participants with baseline assessment who were stroke free and without a dementia diagnosis. [c] APOE4 carrier = at least 1 APOE 4 allele. [d] Hypertension/diabetes/high cholesterol/CVD/depression = identified at baseline or has a history of said condition. [e] Smoking = ever smoked (past or current). [f] Alcohol = ≥ 1 drink per week. [g] Physical activity = moderate or vigorous activity at least once per week.

© 2024 Lo JW et al. *JAMA Network Open.*

eTable 10. Baseline Characteristics of Participants Followed up Until the Last Assessment Versus Participants Who Dropped Out

Characteristics	Followed up (n=8573)	Dropped out (n=12287)	p-value	Cohen's d/ Cohen's h [a]
Age	69.5 (6.9)	75.3 (7.8)	<0.001	0.79
Female	4893 (57%)	7368 (60%)	<0.001	0.06
Education (in years)	10.3 (4.8)	9.9 (4.9)	<0.001	0.08
APOE4 carrier	1148 (21%)	1611 (21%)	0.62	0
BMI	27.1 (4.9)	26.9 (5.2)	0.095	0.02
Systolic blood pressure	139.6 (19.1)	142.0 (20.8)	<0.001	0.12
Hypertension	5762 (67%)	8611 (71%)	<0.001	0.087
Diabetes	1340 (16%)	2249 (18%)	<0.001	0.053
High cholesterol	2662 (32%)	3653 (38%)	<0.001	0.13
CVD	1226 (15%)	2212 (19%)	<0.001	0.11
Smoking (ever)	3479 (41%)	5407 (44%)	<0.001	0.071
Alcohol				
< 1 drink/week	4458 (53%)	6468 (58%)	<0.001	0.10
≥ 1 drink/week	1786 (21%)	1413 (13%)		0.21
≥ 2 drinks/week	2236 (26%)	3314 (30%)		0.089
Physically active				
Minimally active	1390 (23%)	1774 (25%)	<0.001	0.047
Moderately active	3027 (51%)	3996 (57%)		0.12
Vigorous active	1524 (26%)	1298 (18%)		0.19
Depression	1259 (15%)	2651 (23%)	<0.001	0.20
Baseline cognition, z-scores				
MMSE	0.24 (1.09)	-0.15 (1.16)	<0.001	0.35
Global cognition	0.38 (0.99)	-0.07 (1.02)	<0.001	0.45
Processing speed	0.31 (1.06)	-0.034 (1.11)	<0.001	0.32
Memory	0.14 (1.03)	-0.22 (1.09)	<0.001	0.21
Language	0.16 (1.06)	-0.064 (1.07)	<0.001	0.21
Executive function	0.43 (1.06)	0.013 (1.14)	<0.001	0.38

Note. Figures are presented as n (%) or mean (SD).
[a] For both Cohen's d and Cohen's h, values of 0.2, 0.5 and 0.8 are taken to represent small, medium, and large differences between groups in means or proportions.

© 2024 Lo JW et al. *JAMA Network Open.*

eTable 11. Examination of Demographic and Vascular Risk Factor Individually in the Unadjusted Model

Characteristics (n)	Effect size (95% CI)	p-value
Sex (10814)	-0.12 (-0.16, -0.080)	**<0.001**
Age (at baseline; 10814)	-0.066 (-0.070, -0.063)	**<0.001**
Education level, in years (10814)	0.086 (0.080, 0.091)	**<0.001**
Ethno-racial group (10814)		
White participants	(reference)	
Black (American) participants	-0.50 (-0.60, -0.41)	**<0.001**
Asian (90% Chinese) participants	-0.28 (-0.53, -0.041)	**0.022**
African participants	-0.20 (-0.87, 0.48)	0.57
Hispanic (American) participants	-0.093 (-0.30, 0.11)	0.38
Other	-0.85 (-1.12, -0.58)	**<0.001**
Study entry period (10814) [a]		
1990 – 1999	(reference)	
2000 – 2009	0.30 (0.19, 0.41)	**<0.001**
After 2010	0.59 (0.43, 0.74)	**<0.001**
**Vascular risk factors**		
BMI (9318)	0.0034 (-0.0009, 0.0077)	0.12
APOE ε4 carrier (9425)	-0.047 (-0.093, -0.0015)	**0.043**
Blood pressure		
Systolic (9547)	-0.0029 (-0.0040, -0.0018)	**<0.001**
Diastolic (9547)	0.0016 (-0.00042, 0.0036)	0.12
Hypertension (10771)	-0.14 (-0.18, -0.10)	**<0.001**
Diabetes (10772)	-0.23 (-0.29, -0.17)	**<0.001**
High cholesterol (9478)	0.047 (0.0031, 0.090)	**0.036**
Cardiovascular disease (10358)	-0.12 (-0.17, -0.073)	**<0.001**
Ever smoker (10783)	0.024 (-0.015, 0.063)	0.23
Alcohol use (10242) [a]		
Nil/minimal	(reference)	
≥ 1 drink/week	0.11 (0.045, 0.17)	**0.001**
≥ 2 drink/week	0.18 (0.13, 0.23)	**<0.001**
Physical activity (9505) [a]		
Minimal	(reference)	
Moderate	0.11 (0.055, 0.16)	**<0.001**
Vigorous	0.22 (0.16, 0.28)	**<0.001**
Depression (10216)	-0.29 (-0.34, -0.24)	**<0.001**

Note. n = participants with available data for global cognition. All characteristics represent measurements at study baseline or a medical history. Bold p-values indicate significance at the 0.1 level and the associated factor was chosen to be included in the adjusted model. The unadjusted model included time in study (TIS), stroke, and time since stroke (TSS).

[a] The three categories in each of these factors were separately collapsed into two for the adjusted model to allow convergence in the multiple imputation of the covariates (after 2000 vs before; ≥1 drink/week vs <1 drink/week; moderate and vigorous activity vs minimal).

© 2024 Lo JW et al. *JAMA Network Open.*

eTable 12. Missing Data on Covariates Included in the Adjusted Model

	Complete data	Imputed data
Diabetes	20735	125 (0.60%)
Hypertension	20746	114 (0.55%)
Depression	19755	1105 (5.3%)
Alcohol	19675	1185 (5.7%)
CVD	19779	1081 (5.2%)
Systolic blood pressure	18904	1956 (9.4%)
High cholesterol	17910	2950 (14%)
APOE4	13096	7764 (37%)
Physical activity	13009	7851 (38%)

© 2024 Lo JW et al. *JAMA Network Open.*

eTable 13. Unadjusted Estimates of Cognitive Changes in Global Cognition

**Measure (model variable [a])**	**All participants** (n=10,814) [b]	**Stroke only** (n=388)	**No stroke only** (n=10,426)
	**Coefficient (95% CI); p-value**	**Coefficient (95% CI); p-value**	**Coefficient (95% CI); p-value**
Slope without incident stroke (TIS; SD/y)	-0.048 (-0.050, -0.046); <0.001	-0.043 (-0.059, -0.028); <0.001	-0.043 (-0.045, -0.041); <0.001
Acute effect of stroke on cognitive level (stroke; SD)	-0.27 (-0.35, -0.19); <0.001	-0.28 (-0.39, -0.18); <0.001	NA
Difference in post-stroke slope relative to TIS (TSS; SD/y)	-0.039 (-0.058, -0.019); <0.001	-0.031 (-0.055, -0.0062); 0.014	NA

[a] Additional interpretation of model coefficient: TIS (time in study) = rate of decline over stroke-free trajectory; stroke = difference in intercepts between stroke-free and post-stroke trajectories when TSS (time since stroke) = 0; TSS = effect of stroke on rate of decline.

[b] All participants (with global cognition data) were included in the estimate of the slope without a previous stroke; post-stroke decline was estimated in those with an incident stroke (n=388).

[c] The stroke variable from the model estimated the difference in intercepts between two functions at the time of stroke and can be interpreted as acute or short-term change in the level of cognition after stroke.

© 2024 Lo JW et al. *JAMA Network Open.*

eTable 14. Mean Values of Covariates Included the Adjusted Model

Covariates	Mean value[a]
Age at baseline (years)	75.5
Male sex (%)	41%
Education (years)	11.1
Ethno-racial groups (%) [b]	
White participants	81% [b]
Black (American) participants	6.3%
Asian (90% Chinese) participants	6.7%
African participants	4.1%
Hispanic (American) participants	1.2%
Other	0.3%
Study entry period – 2000s and 2010s (%)	71%
Diabetes (%)	15%
Hypertension (%)	71%
CVD (%)	25%
High cholesterol (%)	50%
Systolic blood pressure (mmHg)	138
APOE4 allele carrier (%)	21%
Depressive symptoms (%)	19%
Physical activity – moderate and vigorous activity at least once a week (%)	78%
Alcohol use – 1 or more drinks per week (%)	51%

[a] Mean values of the subsample with global cognition data at baseline.

[b] For simplicity, we estimated predicted cognition scores based on someone who was White.

© 2024 Lo JW et al. *JAMA Network Open.*

eTable 15. Examination of Difference in Cognitive Trajectories Before Stroke Compared With Cognitive Trajectories in Those Without Stroke

Measure	Coefficient (95% CI)	p-value
**Global cognition** (n=10814)		
TIS [a] x group status	0.0080 (-0.0067, 0.023)	0.29
Group status [b]	-0.0035 (-0.10, 0.097)	0.95
**Memory** (n=19322)		
TIS [a] x group status	-0.010 (-0.026, 0.0057)	0.21
Group status [b]	-0.019 (-0.088, 0.050)	0.59
**Language** (n=10453)		
TIS [a] x group status	0.013 (-0.0028, 0.028)	0.096
Group status [b]	-0.023 (-0.12, 0.069)	0.62
**Processing speed** (n=10824)		
TIS [a] x group status	0.0064 (-0.011, 0.024)	0.48
Group status [b]	0.060 (-0.050, 0.17)	0.29
**Executive function**		
TIS [a] x group status	0.015 (-0.0034, 0.033)	0.11
Group status [b]	-0.043 (-0.16, 0.072)	0.46
**MMSE** (n=19835)		
TIS [a] x group status	0.0080 (-0.0071, 0.023)	0.30
Group status [b]	0.017 (-0.046, 0.081)	0.60

The analysis was restricted to the period without a previous stroke for all participants. The model adjusted for baseline age, sex, education, ethno-racial group, study entry period, diabetes, hypertension, CVD, high cholesterol, systolic blood pressure, APOE4, depression, physical activity, and alcohol use. MI was used to impute missing covariates.

[a] TIS=time in study and represents the rate of change in cognition over the period without a previous stroke.

[b] The Group status variable represents difference in the level of cognition between the two groups.

© 2024 Lo JW et al. *JAMA Network Open.*

eTable 16. Sensitivity Analyses
1) complete data only; 2) restrict time since stroke (TSS) to ≥ 1 year; 3) exclude studies with > 50% loss to follow-up

**Measure (model variable)** [a]	**Sensitivity analysis 1**[b] N=8,076 (7 studies)	**Sensitivity analysis 2** N=10,812 (8 studies)	**Sensitivity analysis 3** [c] N=6,478 (5 studies)
	**Coefficient (95% CI); p-value**	**Coefficient (95% CI); p-value**	**Coefficient (95% CI); p-value**
Slope without incident stroke (TIS; SD/y)	-0.050 (-0.052, -0.048); <0.001	-0.049 (-0.051, -0.047); <0.001	-0.046 (-0.048, -0.043); <0.001
Acute effect of stroke on cognitive level (stroke; SD)	-0.31 (-0.40, -0.21); <0.001	-0.25 (-0.34, -0.17); <0.001	-0.26 (-0.38, -0.15); <0.001
Difference in post-stroke slope relative to TIS (TSS; SD/y)	-0.046 (-0.068, -0.023); <0.001	-0.038 (-0.057, -0.018); <0.001	-0.063 (-0.095, -0.032); 0.003

Note. Global cognition z-scores were the outcome. The adjusted models included baseline age, sex, education, ethno-racial group, study entry period, diabetes, hypertension, CVD, high cholesterol, systolic blood pressure, APOE4, depression, physical activity, and alcohol use.

[a] Additional interpretation of model coefficients: TIS (time in study)=rate of decline over stroke-free trajectory; stroke=difference in intercepts between stroke-free and post-stroke trajectories when TSS (time since stroke)=0; TSS=effect of stroke on rate of decline.

[b] EPIDEMCA did not collect data on CVD and hence in the complete data analysis (sensitivity analysis 1), the study was not included. EPIDEMCA also has >50% loss to follow and was therefore excluded in sensitivity analysis 3.

[c] The excluded studies with >50% loss to follow up (and had global cognition scores) were EAS, EPIDEMCA, and ESPRIT. MYHAT had a long follow-up schedule with 13 waves, and they had <50% followed up at the last wave, but >50% at wave 6, therefore we did not exclude this study in the sensitivity analysis. See eTable 1 for full study names.

© 2024 Lo JW et al. *JAMA Network Open.*

eTable 17. Adjusted Estimates of Changes in Cognitive Function in the 4 Cognitive Domains and MMSE Among all Participants

**Measure (model variable)** [a]	**Memory**	**Language**	**Processing speed**	**Executive function**	**MMSE**
N[b]	19327 (13 studies)	10,455 (9 studies)	10,824 (8 studies)	10,166 (7 studies)	19,835 (12 studies)
	**Coefficient (95% CI); p-value**	**Coefficient (95% CI); p-value**	**Coefficient (95% CI); p-value**	**Coefficient (95% CI); p-value**	**Coefficient (95% CI); p-value**
Slope without incident stroke (TIS; SD/y)	-0.031 (-0.034, -0.029); <0.001	-0.043 (-0.045, -0.040): <0.001	-0.037 (-0.040, -0.035); <0.001	-0.053 (-0.055, -0.051); <0.001	-0.019 (-0.021, -0.017); <0.001
Acute effect of stroke on cognitive level (stroke; SD)	-0.21 (-0.28, -0.14): <0.001	-0.22 (-0.30, -0.14); <0.001	-0.17 (-0.26, -0.080); <0.001	-0.19 (-0.29, -0.083); <0.001	-0.36 (-0.43, -0.29); <0.001
Difference in post-stroke slope relative to TIS (TSS; SD/y)	-0.015 (-0.037, 0.007): 0.17	-0.020 (-0.039, -0.0010); 0.040	-0.055 (-0.076, -0.035); <0.001	-0.030 (-0.053, -0.0074); 0.009	-0.0072 (-0.024, 0.0094); 0.40
Total post-stroke slope (TIS+TSS; SD/y)	-0.047 (-0.068, -0.025); <0.001	-0.063 (-0.082, -0.044); <0.001	-0.093 (-0.11, -0.072); <0.001	-0.083 (-0.11, -0.060); <0.001	-0.027 (-0.043, -0.010); 0.002

Note. Cognitive scores were standardized scores (SD). TIS=time in study; TSS=time since stroke. The models adjusted for age, sex, education, ethno-racial groups, study entry period, diabetes, hypertension, high cholesterol, systolic blood pressure, CVD, APOE4 carrier status, depressive symptoms, physical activity, and alcohol use.
[a] Additional interpretation of model coefficients: TIS (time in study)=rate of decline over stroke-free trajectory; stroke=difference in intercepts between stroke-free and post-stroke trajectories when TSS (time since stroke)=0; TSS=effect of stroke on rate of decline.
[b] N is based on studies having conducted neuropsychological tests in that domain, see eTable 6 for details.

© 2024 Lo JW et al. *JAMA Network Open.*

eTable 18. Subgroup Analyses in Stroke Group and No-Stroke Group in Cognitive Domains and MMSE

Measure (model variable) [a]	Memory	Language	Processing speed	Executive function	MMSE
**Stroke only**	N=887	N=466	N=385	N=347	N=1,002
Slope without incident stroke (TIS; SD/y)	-0.047 (-0.062, -0.031); <0.001	-0.049 (-0.063, -0.036); <0.001	-0.037 (-0.054, -0.021); <0.001	-0.050 (-0.067, -0.031); <0.001	-0.023 (-0.036, -0.010); 0.001
Acute effect of stroke on cognitive level (stroke; SD)	-0.17 (-0.25, -0.079); <0.001	-0.20 (-0.30, -0.10); <0.001	-0.18 (-0.30, -0.067); 0.002	-0.20 (-0.33, -0.064); 0.004	-0.37 (-0.45, -0.28); <0.001
Difference in post-stroke slope relative to TIS (TSS; SD/y)	-0.0020 (-0.028, 0.024); 0.88	-0.014 (-.038, 0.0090); 0.23	-0.054 (-0.081, -0.028); <0.001	-0.031 (-0.060, -0.0016); 0.038	-0.007 (-0.028, 0.014); 0.51
**No stroke only**	N=18,237	N=9989	N=10,439	N=9,819	N=18,833
Slope (SD/y)	-0.031 (-0.033, -0.029); <0.001	-0.042 (-0.045, -0.040); <0.001	-0.037 (-0.040, -0.035); <0.001	-0.053 (-0.056, -0.051); <0.001	-0.019 (-0.021, -0.017); <0.001

Note. All outcomes were standardized scores (SD). The models adjusted for age, sex, education, ethno-racial groups, study entry period, diabetes, hypertension, high cholesterol, systolic blood pressure, CVD, APOE4 carrier status, depressive symptoms, physical activity, and alcohol use.

[a] Additional interpretation of model coefficients: TIS (time in study)=rate of decline over stroke-free trajectory; stroke=difference in intercepts between stroke-free and post-stroke trajectories when TSS (time since stroke)=0; TSS=effect of stroke on rate of decline.

© 2024 Lo JW et al. *JAMA Network Open.*

eTable 19. Examination of Moderating Effects in the Trajectory of Poststroke Cognitive Function

**Age**	**Coefficient (95% CI)**	**p-value**
Age x TSS<br>Age x acute effect<br>Age x TIS<br><br>≥72 x TSS<br>≥72 x acute effect<br>≥72 x TIS	NS<br>0.013 (0.0017, 0.023)<br>-0.0030 (-0.0059, -0.0052)<br><br>NS<br>0.12 (-0.025, 0.26)<br>-0.031 (-0.035, -0.027)	-<br>**0.024**<br>**<0.001**<br><br>-<br>0.11<br><0.001
**Education (in years)**		
Education x TSS<br>Education x acute effect<br>Education x TIS	NS<br>-0.011 (-0.027, 0.004)<br>~0.00 (-0.0005, 0.0006)	-<br>0.14<br>0.90
**Diabetes**		
Diabetes x TSS<br>Diabetes x acute effect<br>Diabetes x TIS	NS<br>0.17 (-0.016, 0.34)<br>-0.0059 (-0.012, -0.0001)	-<br>0.052<br>0.0045
**Hypertension**		
Hypertension x TSS<br>Hypertension x acute effect<br>Hypertension x TIS	0.035 (-0.0044, 0.075)<br>NS<br>-0.0082 (-0.012, -0.0041)	0.081<br>-<br><0.001
**APOE4 carrier**		
APOE4 x TSS<br>APOE 4 x acute effect<br>APOE4 x TIS	-0.049 (-0.10, 0.045)<br>NS<br>-0.014 (-0.019, -0.0090)	0.072<br>-<br><0.001
**Depressive symptoms**		
Depress x TSS<br>Depress x acute effect<br>Depress x TIS	NS<br>-0.11 (-0.32, 0.072)<br>-0.0074 (-0.012, -0.0024)	-<br>0.24<br>0.004

© 2024 Lo JW et al. *JAMA Network Open.*

Not significant (at p<0.2) for the interactions between both TSS and acute effect with the following factors: sex, high cholesterol, cardiovascular disease, smoking

Interactions with TIS were significant:
TIS x cholesterol: -0.007 (-0.011, -0.003); <0.001
TIS x CVD: -0.005 (-0.010, -0.0005); 0.029
TIS x smoking: -0.006 (-0.010, -0.003); 0.001

Note. Global cognition was used as the outcome (n=10,814 from 8 studies). NS=not significant at p<0.2 and taken out of the model. Only results p<0.2 were kept in the model and shown above. TIS= time in study and represents the rate of change over time when there was no previous stroke in all participants; model coefficient of TSS denotes the post-stroke slope compared with TIS. Acute effect = model estimated acute change at time of stroke. Each model adjusted for baseline age, sex, education, ethno-racial group, study entry period, diabetes, hypertension, high cholesterol, systolic blood pressure, CVD, APOE4 status, depression, physical activity, and alcohol use.

© 2024 Lo JW et al. *JAMA Network Open.*

eTable 20. Stratified Analyses for Age Groups With Global Cognition as the Outcome

**< 72 years** N=4652 (8 studies)		**≥ 72 years** N=6162 (7 studies)	
Acute effect	-0.33 (-0.47, -0.19); <0.001	Acute effect	-0.19 (-0.29, -0.092); <0.001
TSS	-0.025 (-0.055, 0.005); 0.11	TSS	-0.042 (-0.066, -0.017); 0.001
TIS	-0.034 (-0.036, -0.031); <0.001	TIS	-0.063 (-0.066, -0.061); <0.001
Intercept [a]	0.38 (0.12, 0.64); 0.005	Intercept [a]	-0.10 (-0.46, 0.25); 0.50

TIS= time in study and represents the rate of change over time when there was no previous stroke in all participants; TSS denotes the rate of change in global cognition scores (SD/year) after stroke compared with TIS. Acute effect = acute effect of stroke on cognitive level.

[a] Intercept values are based on model using covariates centered at the mean.

eTable 21. Stratified Analyses for Diabetes Groups With Global Cognition as the Outcome

**With diabetes** N=1059 (7 studies)		**Without diabetes** N=7017 (7 studies)	
Acute effect	-0.12 (-0.34, 0.093); 0.26	Acute effect	-0.35 (-0.46, -0.24); <0.001
TSS	-0.078 (-0.13, -0.025); 0.004	TSS	-0.037 (-0.062, -0.012); 0.004
TIS	-0.056 (-0.063, -0.050); <0.001	TIS	-0.049 (-0.051, -0.046); <0.001
Intercept [a]	-0.34 (-0.83, 0.15); 0.17	Intercept [a]	-0.11 (-0.50, 0.27); 0.57

Note. MI was not used since sample varies between different imputations. TIS= time in study and represents the rate of change over time when there was no previous stroke in all participants; TSS denotes the rate of change in global cognition scores (SD/year) after stroke compared with TIS. Acute effect = acute effect of stroke on cognitive level.

[a] Intercept values are based on model using covariates centered at the mean.

© 2024 Lo JW et al. *JAMA Network Open.*

eFigure 1. Follow-up Schedule for Each Contributing Study

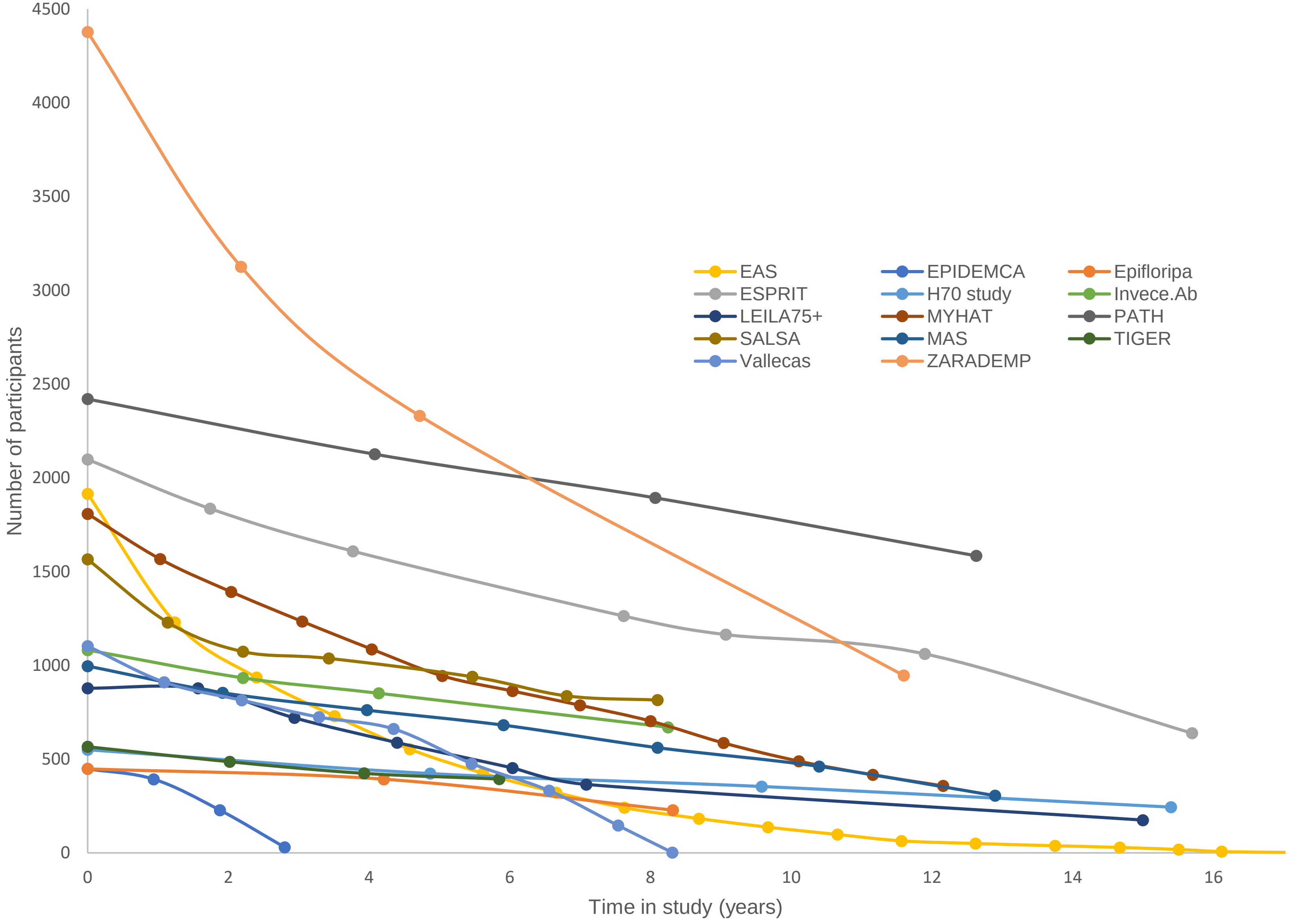

© 2024 Lo JW et al. *JAMA Network Open.*

eFigure 2. Predicted Values of Global Cognition for Participants Aged Younger Than 72 and 72 Years and Older

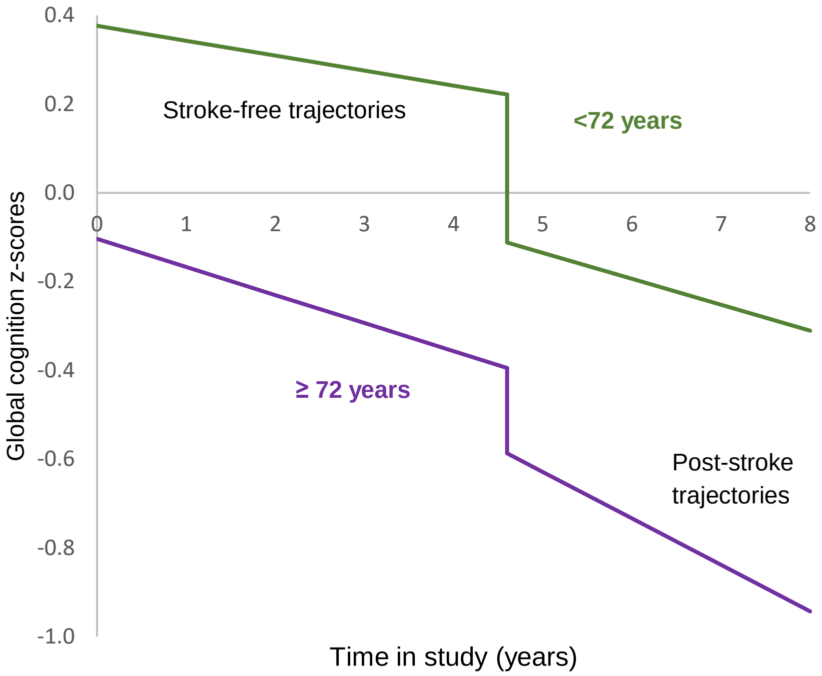

Note. Predicted values of global cognition scores were calculated for common values of covariates at baseline (based on sample with global cognition data). The estimates were calculated for stroke occurring at 4.6 years into the study.

© 2024 Lo JW et al. *JAMA Network Open.*

eFigure 3. Predicted Values of Global Cognition for Participants With and Without Diabetes

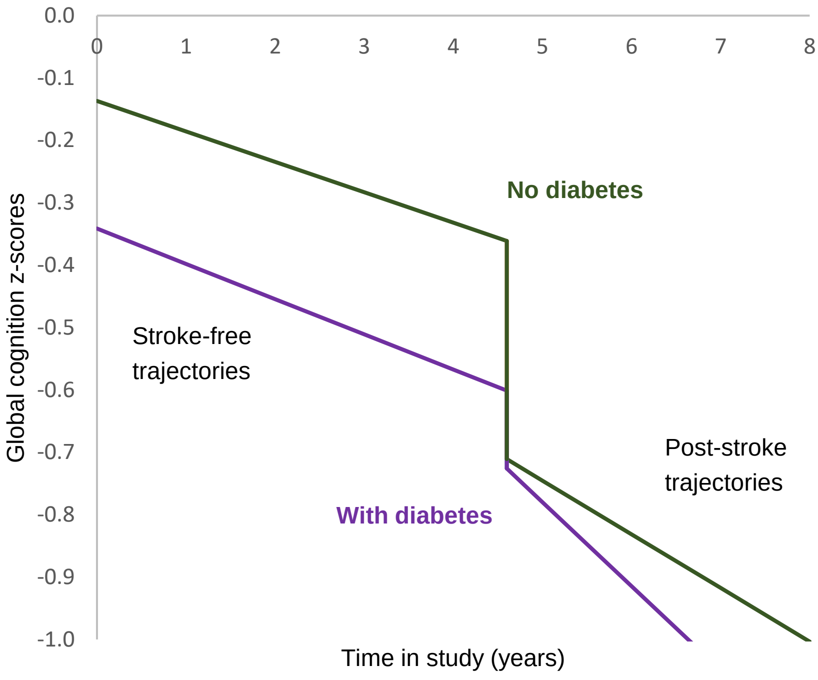

Note. Predicted values of global cognition scores were calculated for common values of covariates at baseline (based on sample with global cognition data). The estimates were calculated for stroke occurring at 4.6 years into the study.

© 2024 Lo JW et al. *JAMA Network Open.*

eFigure 4. Predicted Values of Global Cognition Among all Participants and in Subgroups, With 95% CI

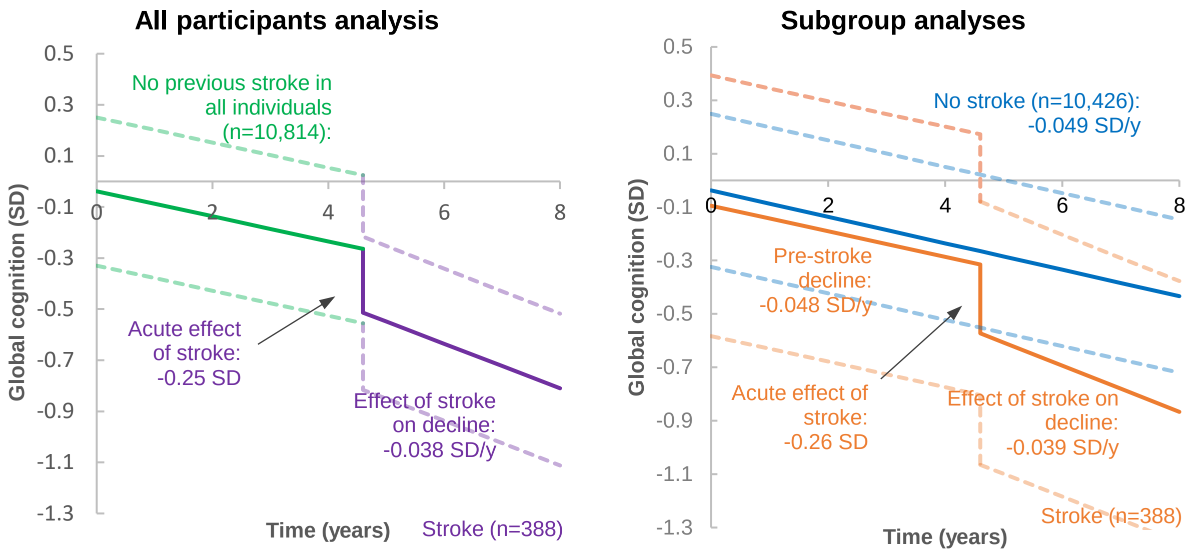

Note. Predicted values of cognition scores were calculated for common values of covariates at baseline and for stroke occurring at 4.6 years into the study. Common values were based on subsample with global cognition data, see eTable 14. The large size of the CIs is primarily due to the large standard error of the intercept (when TIS = 0).

© 2024 Lo JW et al. *JAMA Network Open.*

eFigure 5. Predicted Values of Cognitive Function in Each Domain and MMSE Among all Participants, With 95% CI

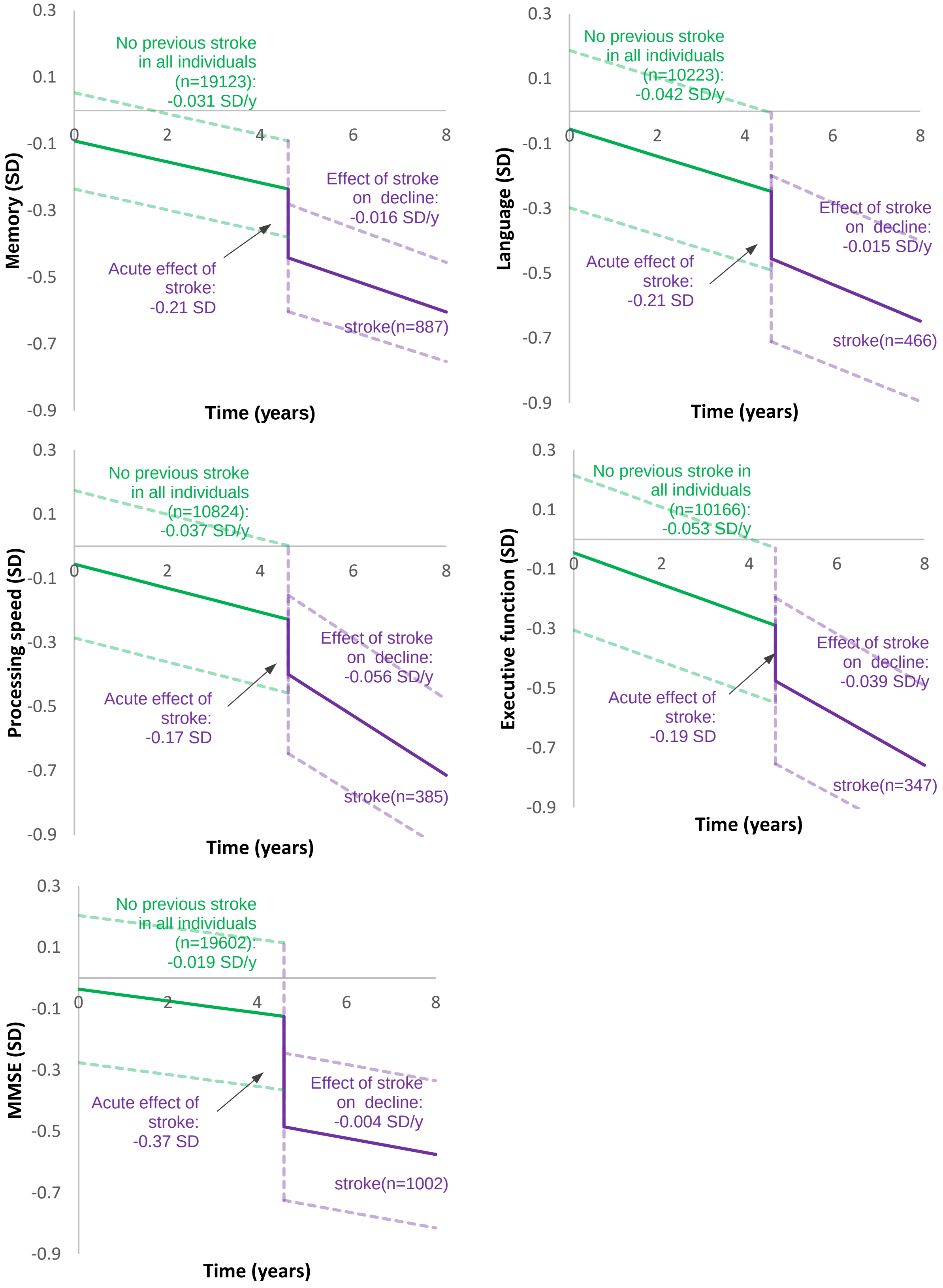

Note. Dotted lines denote 95% confidence intervals (CI). Predicted values of cognition scores were calculated for common values of covariates at baseline and for stroke occurring at 4.6 years into the study. Common values were based on subsample with global cognition data, see eTable 14. The large size of the CIs is primarily due to the large standard error of the intercept (when TIS = 0).

© 2024 Lo JW et al. *JAMA Network Open.*
