# Supplementary material for: Trajectory of Cognitive Decline Before and After Stroke in 14 Population Cohorts
Source: JAMA Netw Open. 2024 Oct 2;7(10):e2437133. doi: 10.1001/jamanetworkopen.2024.37133 (PMC11447567; doi:10.1001/jamanetworkopen.2024.37133)
Supplement: Supplement 2. — Data Sharing Statement [file jamanetwopen-e2437133-s002.pdf]

Data Sharing Statement

Lo. Trajectory of Cognitive Decline Before and After Stroke in 14 Population Cohorts. *JAMA Netw Open*. Published October 02, 2024. doi:10.1001/jamanetworkopen.2024.37133

Data
Data available: No

Additional Information
Explanation for why data not available: Individual patient data from included studies may be accessed upon an application via the COSMIC consortium.
